# Transition Metal Dichalcogenides for the Application of Pollution Reduction: A Review

**DOI:** 10.3390/nano10061012

**Published:** 2020-05-26

**Authors:** Xixia Zhang, Sin Yong Teng, Adrian Chun Minh Loy, Bing Shen How, Wei Dong Leong, Xutang Tao

**Affiliations:** 1 State Key Laboratory of Crystal Materials, Shandong University, Jinan 250100, China; txt@sdu.edu.cn; 2 Central European Institute of Technology, Brno University of Technology, Purkynova 656/123, 612 00 Brno, Czech Republic; 3 Institute of Process Engineering & NETME Centre, Brno University of Technology, Technicka 2896/2, 616 69 Brno, Czech Republic; Sin.Yong.Teng@vut.cz; 4 Department of Chemical Engineering, Monash University, Clayton, Melbourne 3800, Australia; adrian.loy@monash.edu; 5 Research Centre for Sustainable Technologies, Faculty of Engineering, Computing and Science, Swinburne University of Technology, Jalan Simpang Tiga, Kuching 93350, Malaysia; bshow@swinburne.edu.my; 6 Department of Chemical and Environmental Engineering, University of Nottingham, Semenyih 43500, Malaysia; ebxwl1@nottingham.edu.my

**Keywords:** transition metal dichalcogenide (TMDCs) nanomaterials, layered materials, nanocatalysis, gas cleaning, catalysis, pollution reduction, emission control

## Abstract

The material characteristics and properties of transition metal dichalcogenide (TMDCs) have gained research interest in various fields, such as electronics, catalytic, and energy storage. In particular, many researchers have been focusing on the applications of TMDCs in dealing with environmental pollution. TMDCs provide a unique opportunity to develop higher-value applications related to environmental matters. This work highlights the applications of TMDCs contributing to pollution reduction in (i) gas sensing technology, (ii) gas adsorption and removal, (iii) wastewater treatment, (iv) fuel cleaning, and (v) carbon dioxide valorization and conversion. Overall, the applications of TMDCs have successfully demonstrated the advantages of contributing to environmental conversation due to their special properties. The challenges and bottlenecks of implementing TMDCs in the actual industry are also highlighted. More efforts need to be devoted to overcoming the hurdles to maximize the potential of TMDCs implementation in the industry.

## 1. Introduction

Transition metal dichalcogenides (TMDCs) are a large family of two-dimensional (2D) layered materials, which are scientifically interesting and industrially important. These materials have attracted tremendous attention because of the unique structural features and interesting properties, such as optoelectronics, electronics, mechanical, optical, catalytical, energy-storage, thermal, and superconductivity properties [1,2,3,4,5,6,7]. TMDCs are the compounds of the chemical formula MX_2_, where M is a transition metal element of groups IV-VII B (Mo, W, V, Nb, Ta, Ti, Zr, Hf, Tc, and Re) and X is a chalcogen element (S, Se, and Te). The X-M-X unit layer consists of three atomic layers, in which one centre atom layer (M) is sandwiched between two chalcogen atom layers (X). TMDCs occupy the layered structures, which resemble that of graphite. The interlayers are stacked by weak van der Waals force, leading to the formation of monolayers or nanolayers from the bulk materials via exfoliation [8]. Different stacking of the layers along c-axis determines polymorphic crystal structures in TMDCs, and the common phases are 1T, 2H, 3R, and Td phases (T—trigonal, H—hexagonal, R—rhombohedral, and Td—distorted octahedral) [9].

There are more than 40 different TMDC types, including metals (such as TiS_2_ and VSe_2_), superconductors (such as TaS_2_ and NbS_2_), semimetals (such as MoTe_2_ and WTe_2_), and semiconductors (MoS_2_, MoSe_2_, WS_2_, and WSe_2_). TMDCs exhibit interesting band structures with tunable bandgaps. The bandgap is one of the most important factors in 2D materials for determining the properties and applications. For instance, graphene is a semimetal with zero bandgap, which limits its applications in electronics and photo-electronics. TMDCs exhibit variable bandgaps from 0 to 3 eV, which can be tuned by thickness [10], defects [11], dopants [12], and mechanical deformations (by applying the tensile strain or compressive strain) [13,14]. The most studied semiconducting TMDCs (e.g., MoS_2_, MoSe_2_, WS_2_, and WSe_2_) have shown typical features in electronic structures. The bandgap increases with the decreasing thickness and it possesses the transition from indirect in the bulk crystals to direct in the monolayers [10,15]. For instance, the indirect bandgap of −1.29 eV will be changed to a direct bandgap of −1.8 eV when bulk MoS_2_ is down to a monolayer [16].

Benefiting from their unique crystal structures and electronic structures, TMDCs have shown great potential in various fields, including electronics/optoelectronics [1,17], catalysis [18], energy storage [19] and conversion [20], sensing [21,22], and so on. The application of TMDCs in pollution reduction is a compelling research topic. The increasing environmental pollution issue has been one of the most serious problems on Earth. Enormous efforts have been made to search the efficient and low-cost methods for addressing the environmental pollution issue. TMDCs may be a kind of promising materials for tackling these problems with several advantages. Firstly, TMDCs have a high surface-to-volume ratio. They offer more effective active sites on the surface, as well as abundant unsaturated surface sites. Thus, the layered TMDCs are excellent platforms for the anchor of semiconductor nanoparticles in various photocatalytic applications [23,24]. Due to their high surface-to-volume ratio, TMDCs are extremely sensitive to the surrounding atmosphere and can be utilized in toxic gas sensing and adsorption. Secondly, TMDCs have tunable bandgaps, which enhances the photocatalytic performance in nanocomposite by offering appropriate bandgap and band alignment [25]. Thirdly, defect engineering can be easy to implement in 2D materials, which have been confirmed to be an efficient method for intensifying the catalytic activities in TMDCs [26,27,28,29]. Lastly but importantly, there is a large variety for TMDCs (about 40 kinds) and they have an abundant amount in nature or can be synthesized [9]. So far, MoS_2_, WS_2_, MoSe_2_, and ReS_2_ have been naturally found [30,31,32]. Specifically, MoS_2_ exists as molybdenite in nature and is the main source of molybdenum with a large amount [33]. The main metals (W and Mo) in TMDCs are both abundant, cheap, and widely used in industry [34]. TMDCs can be prepared by using various techniques, such as chemical vapor deposition (CVD) [35,36], chemical vapor transport (CVT) [37,38], flux growth method [39], hydrothermal synthesis [40], Langmuir−Schaefer deposition [41], etc. In addition, the top-down exfoliated method can be also used to fabricate few-layer TMDCs from bulk crystals, e.g., mechanical exfoliations and liquid phase exfoliations [42,43,44]. With increasing interests in TMDCs for applications, we aim to prepare an overview of the recent progress of TMDCs in reducing the environmental pollution. We will summarize the representative efforts, including gas adsorption and removal, gas sensing, wastewater treatment, fuel cleaning, CO_2_ valorization, and conversion.

## 2. Gas Adsorption and Removal

**Figure 1:**
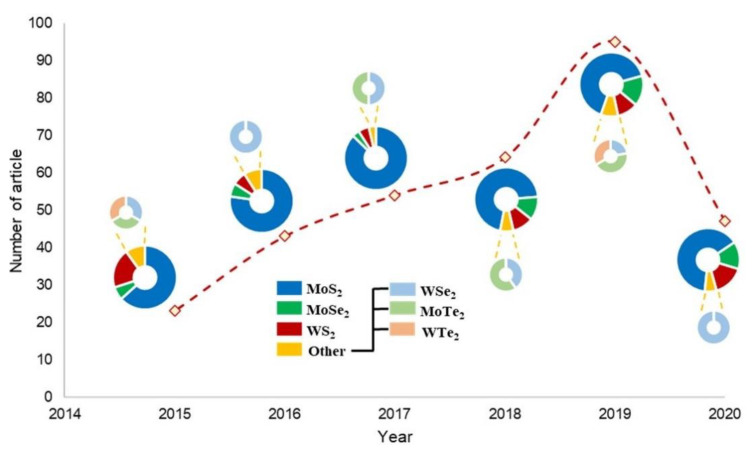
The number of research articles that applied transition metal dichalcogenides (TMDCs) in gas adsorption application between the year 2015 to April 2020, where pie charts represent the utilization percentage of various types of TMDC. Queried from Scopus Database [48]. Copyright Elsevier B.V., 2020.

**Figure 2:**
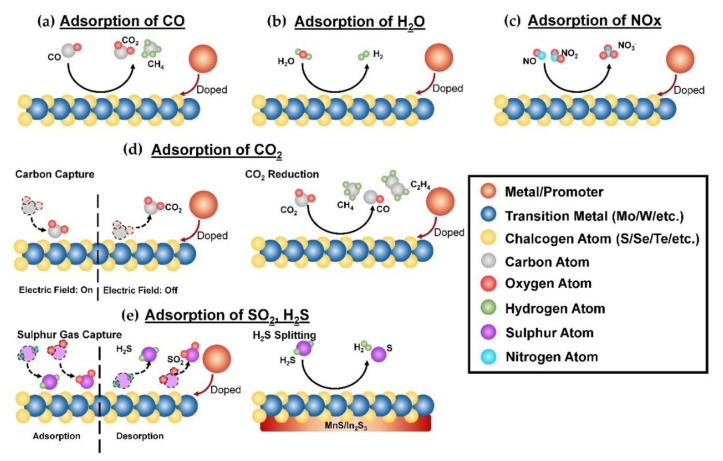
Schematic diagrams for the adsorption processes of (**a**) CO, (**b**) H_2_O, (**c**) CO_2_, (**d**) sulphur content, and (**e**) NO*_x_* with the use of TMDCs.

In recent years, TMDCs have been used as gas removal (via adsorption) in pollution reduction. Figure 1 presents the number of research articles that involved the use of TMDCs in gas adsorption applications. The increasing trend (the total number of related articles published in the year 2019 is more than three-fold than that of the year 2015) indicates the potential of TMDCs in gas removal processes. Based on the bibliology search, MoS_2_ is still the most widely studied TMDCs among the TMDCs considered in this review. However, researchers started to realize that there are other TMDCs (e.g., MoSe_2_ and WS_2_) that offer better performances as compared to MoS_2_ (e.g., greater charge storage ability [45]). This further led to a gradual increase in research articles that studied the use of other potential TMDCs, since the year 2018. Figure 2 outlines the schematic diagrams of each gas adsorption process. In general, the mechanism of these adsorption processes is mainly driven by the charge transfer process between the adsorbates and the TMDCs-based adsorbent, where the charge movement is dependent on the nature of the gas adsorbate (i.e., oxidizing or reducing) [46]. For instance, carbon monoxide (CO) in Figure 2a is an example of reducing gas. Due to the existence of lone pair on the carbon atom, CO will donate electrons to the TMDC surface, which further cause the CO to be chemisorbed on the surface. Whereas, oxidizing gas such as nitrogen dioxide (NO_2_) in Figure 2c, will uptake the electron from the surface instead (mainly due to the existence of unpaired electron on the nitrogen atom). To note, such electron movement will lead to the deviation in electrical conductivity of the TMDC materials [47]. The following subsections discuss various gas adsorption processes.

### 2.1. Adsorption of Carbon Monoxide (CO)

Carbon monoxide (CO) is by far the most hazardous greenhouse gases, which is 210 times easier to bind with hemoglobin as compared to the oxygen [49]. To date, numerous studies have discovered the use of TMDCs as a catalyst to adsorb CO and further convert it into other products via catalytic reaction. For instance, Li et al. [50] proposed the use of aluminum oxide-doped MoS_2_ as the nanocatalysts to promote the CO methanation process (CO + 3H_2_ → CH_4_ + H_2_O) and enhance the stability of this catalytic reaction (MoS_2_ with 25.6% of Al_2_O_3_ provides the greatest methanation stability). The schematic diagram of this process is shown in Figure 2a. The incorporation of metal-based promoters, in this case, Al_2_O_3_ powder has effectively reduced the aggregation effect between MoS_2_ (lower tendency to pore blockage) [50]. Aside from that, researchers also adopted the density functional theory (DFT) calculation to explore the theoretical potential of each TMDC as the alternative catalyst for CO oxidation (CO + O_2_ → CO_2_ + O) and CO dissociation. For instances, studies showed that metal-doped MoS_2_ (e.g., Au_29_, Cu, Ag, Co, Rh, Ni, Ir, and Fe [51,52,53]) can significantly enhance the O_2_ dissociation (which is the essential step for CO oxidation). On the other hand, the latter five mentioned metals possess the greatest potential as they favor CO dissociation [52]. More recently, application of other TMDCs (e.g., Pt and Au nanoclusters with WSe_2_, where the carbon atom in CO will be strongly adsorbed on the Pt/Au decorated WSe_2_ monolayer [54]; Rh-doped MoSe_2_ [55]), in CO adsorption have also been carried out. Since CO dissociation is the first step of the Fischer–Tropsch process [56], the aforementioned TMDC-based nanocatalysts can be served as the substituent cheap and stable catalyst to convert CO into other valuable liquid hydrocarbons.

### 2.2. Adsorption of Water Vapor (H_2_O)

Water vapor (H_2_O) is the largest contributor to the current global warming issue (i.e., roughly accounted for 60% of the entire warming effect [57]). Numerous works have proposed the use of TMDC-based nanocatalysts to convert H_2_O into clean hydrogen (H_2_) via hydrogen evolution reaction (HER) (see Figure 2b). In general, hydrogen atoms in H_2_O will be adsorbed on the active site of the HER nanocatalysts. With the aid of the doped metal, the dissociation of the O–H bond in H_2_O is enhanced. The gas is then desorbed as H_2_ [58,59]. This can mitigate the warming effect attributed to the water vapor content in the atmosphere, and at the same time, serves as an alternative greenway for hydrogen production. The past two decades ago, MoS_2_ was referred to as one of the most prominent alternatives to substitute the conventional HER catalysts [60]. Nevertheless, the commercialization of TMDC-based HER nanocatalysts is still hindered due to various technical challenges (e.g., poor HER stability under acidic conditions [61,62] and weak intrinsic conductivity [63]). Numerous works have discovered ways to enhance the HER performance of MoS_2_. Just to name a few, these works proposed to improve the HER activity by (i) coating the MoS_2_ with metals (e.g., palladium [64], aluminum [65], gold [66], platinum [67], etc.); (ii) using a novel electrochemical approach to deposit the MoS_2_ nanosheet [68]; (iii) introducing the use promising support for the MoS_2_ nanoparticles (e.g., multi-wall carbon nanotubes [69]); (iv) varying the choice of dispersion media used [70]; and (v) coupling with other electrocatalysts (e.g., molybdenum carbide [71]). On the other hand, some researchers also proposed the use of other TMDCs in HER applications. For instance, Wang et al. [72] proposed to use a low-cost MoSe_2_ nanosheet, which offers decent active sites for HER activity and better HER performance as compared to the aforementioned MoS_2_-based catalysts. On the other hand, Seok et al. [73] have reported the intrinsic activity for HER on MoTe_2_ nanosheet via the first-principle calculation and scanning tunneling microscopy (STM) study. Besides, numerous researchers also successfully developed promising WS_2_-based and WSe_2_-based electrocatalysts recently (e.g., Te–WS_2_ nanosheet by Pan et al. [74]; and Ni-WSe_2_ nanosheet by Kadam et al. [75]).

### 2.3. Adsorption of Carbon Dioxide (CO_2_)

Aside from water vapor, carbon dioxide (CO_2_) is another key contributor to climate change issues (accounting for more than 75% of the total emitted greenhouse gases [76]). To address this issue, some researchers have attempted to apply MoS_2_ nanosheets as an effective membrane [77] and adsorbent [78,79] to separate CO_2_ from the gaseous mixture. Sun et al. [78] reported that the adsorption force between CO_2_ and MoS_2_ varies according to the strength of the applied electric field. Generally, CO_2_ will adsorb on the surface when an electric field is applied and desorb when the electric field is relieved (see Figure 2d). This unique feature makes it become a potential carbon capture media. In addition to the carbon capture process, TMDCs can be modified so that the captured CO_2_ can be catalytically converted into other CO_2_-reduction products. Shi et al. [80] were the first to discover the potential of copper modified MoS_2_ nanosheets in the CO_2_ reduction process. The incorporation of Cu nanoparticles has enhanced the adsorption capability as CO_2_ can now be adsorbed not only on the MoS_2_ nanosheets but also on the doped metal nanoparticles [80] (see Figure 2d). This work found that most of the CO_2_ was converted into CO (i.e., faradaic efficiency (FE) = 33–41%), followed by methane (FE = 7–17%) and with a trace amount of ethylene (FE = 2–3.5%) [80]. More recently, a research project in Korea has discovered the potential of a composite catalyst that encompasses MoS_2_ nanosheets and *n*-type Bi_2_S_3_, in the CO_2_-to-CO photoreduction process [81]. DFT calculations of various modified-TMDC nanosheets (e.g., SnO_2_-loaded MoS_2_ nanosheet [82]; TiO_2_-doped MoS_2_ nanosheets [83]) for the CO_2_ reduction process were also conducted recently. Nevertheless, the research on the potential of other TMDCs in CO_2_ adsorption is still considered scarce. To date, only a few have discovered their potential for the CO_2_ sensor [84,85].

### 2.4. Sulphur Content Removal

Based on the air quality data observed by the NASA Ozone Monitoring Instrument (OMI) satellite, about 60% of the total sulphur emissions in 2018 were anthropogenic emissions [86]. Despite the recent advancement in renewable energy and air treatment technologies have reduced the global sulphur emissions (sulphur emissions in 2015 is about 30% less as compared to that of 1990 [87]), the health impact of sulphur content in the atmosphere can still be severe. Thus, the sulphur emissions (including SO_2_, H_2_S, etc.) must still be monitored and controlled closely. Wei et al. [56], based on the DFT simulation, have explored the adsorption performance of SO_2_ and H_2_S on the Ni-doped MoS_2_ monolayer material. To note, due to the large number of free electrons offered by the doped metals, the overall adsorption capability of the TMDC-based adsorbents are, therefore, gradually enhanced [88]. This study is then extended by other researchers, by testing the effect of other doped metal atoms (e.g., palladium [89], platinum [90], gold [89], and copper [91]). All these studies show that the metal-doped MoS_2_ monolayer materials can offer promising adsorption properties to SO_2_ and H_2_S gases, where both gases are chemisorbed on the surface with strong interactive forces. Similarly, various DFT studies have also been conducted to study the potential of using metal-doped MoSe_2_ nanosheets (e.g., rhodium-doped MoSe_2_ monolayer material [55] and palladium-doped MoSe_2_ monolayer material [92]) and WSe_2_ nanosheets (e.g., N-doped TiO_2_/WSe_2_ nanocomposite [93] and silver-doped WSe_2_ monolayer material [94]) in sulphur content removal applications. Dan et al. [95], on the other hand, studied the photocatalytic H_2_S splitting (H_2_S → H_2_ + S) on the proposed novel composite catalysts, MnS/In_2_S_3_-MoS_2_ based on first-principles calculations. This is an attractive and cost-effective way to generate clean H_2_ that can substitute conventional coal-derived hydrogen production [96]. In other words, this also indirectly contributes to pollution mitigation. The schematic diagram for the aforementioned sulphur removal process is shown in Figure 2e, where the desorption process can be conducted by altering the operating conditions (e.g., temperature and pressure).

### 2.5. Nitrogen Oxide (NO_x_) Removal

Nitrogen oxide (NO*_x_*) has adverse effects on human health and ecosystems. To date, numerous works have revealed the potential of applying TMDCs for NO*_x_* removal purposes. Notably, a novel Cu_2_O-anchored MoS_2_ nanocomposite developed by Yuan et al. [97], can achieve up to 53% NO*_x_* removal. In another research conducted in China, a similar NO removal rate (about 51%) can also be obtained using the MoS_2_-g-C_3_N_4_ nanocomposite [98]. In the same year, Xiong et al. [99] managed to develop a novel (BiO)_2_CO_3_/MoS_2_ photocatalysts that could achieve 50% NO removal for five consecutive cycles. The study was extended by incorporating the use of carbon nanofibers into nanocomposites fabrication [100]. The developed Bi_2_O_2_CO_3_-MoS_2_-CNFs nanocomposites can attain almost 70% of NO removal rate. Under visible light irradiation, the oxidative power offered by the nanocomposites is sufficient to oxidize the NO*_x_* into NO^3−^ (see Figure 2c). Besides, first-principle studies were also conducted to determine the metal-doping effects on the adsorption capabilities on MoS_2_ (e.g., (vanadium, niobium, tantalum)-doped MoS_2_ [101]), MoSe_2_ (e.g., palladium-doped MoSe_2_ [92] and rhodium-doped MoSe_2_ [55]), and WSe_2_ [102] monolayer materials to NO*_x_* gases.

## 3. Gas Sensing for Pollution Reduction

Gas pollution is commonly caused by direct greenhouse gases (CO, CO_2_, N_2_O, CH_4_, Fluorinated Gas, etc.) [103], indirect greenhouse gases (NH_3_, NO*_x_*, H_2_S, etc.) [104], and other traces of toxic gases. These gas pollutants may cause climate change, ozone pollution, and even threaten food security [105] Further adverse effects that are caused by these problems include reduced photosynthesis performances [106], increased chances of respiratory problems [107], and acid rain [108].

Gas sensing can be deployed in multiple locations to act as an environmental monitoring system [109]. More recent applications of gas sensing products include gas monitoring using drones [110], wearable gas sensing devices [111], and the internet of things (IoT) multi-gas sensing modules [112]. For gas sensing TMDC-material in the device, the most common working principle is for adsorption of gas particles towards the TMDCs for a charge transfer, giving a change of resistivity in material, then desorption from the TMDCs [113]. In this field, researchers are constantly searching for TMDC materials that can provide a very low limit of detection (LOD), high sensitivity, short response and recovery time [114,115,116].

### 3.1. NO_x_ Detection

NO*_x_* detection by TMDCs has received much research interest due to its effectiveness and applicability. The measurement of NO*_x_* in environmental monitoring is a common requirement [117] while the design of a perfect NO*_x_* sensor poses some challenges. Traditional SnO_2_ sensors have problems with the dual response towards oxidizing gases and reducing gases, giving low sensitivity when performing measurements in a multi-gas environment [118]. The selectivity of the gas between NO_2_ and NO is also a crucial challenge for NO*_x_* sensors [119].

A popular TMDC material for NO_2_ detection is MoS_2_ [113,120,121]. Earlier works [121] used CVD-grown MoS_2_ to detect NO_2_ gas at room temperature with LOD down to parts per billion (ppb) ranges, however, the recovery time was long. Consequently, a higher temperature was used to accelerate the desorption kinetics of the NO_2_ gas molecule [113,120]. Other researchers also created nanocomposites of TMDCs with materials such as graphene aerogel [122] and reduced graphene oxide (rGO) [123] to improve sensitivity and lower LOD. Pham et al. [115] used a red-light illumination to provide photon energy, which matches the bandgap of the MoSe_2_ sensor. The technique resulted in low LOD (25 ppb) in room temperature conditions with high sensitivity. Moreover, Liu et al. [114] synthesized a flower-like porous SnS_2_ NSs with edge exposed MoS_2_ nanosphere, which resulted in a very fast response time of 2 seconds. Group-10 noble TMDCs such as PtSe_2_ has also received much research attention due to their widely tunable bandgap and excellent performance in gas sensing [124]. From a first-principle study, Sajjad et al. [125] discovered that monolayer PtSe_2_ exhibited lower adsorption energy than MoS_2_ and graphene. For this, Yim et al. [126] used thin films of PtSe_2_ stacked on Si to achieve NO_2_ detection down to 9 ppb. Other TMDC materials such as WS_2_ [122] and MoTe_2_ [127] also showed that they have excellent NO_2_ sensing properties, and more research work is to be expected for different transition metals. A collection of NO_2_ sensing TMDC materials is tabulated in Table 1.

Although NO and NO_2_ are sometimes measured as total NO*_x_*, many academic studies require differentiation between NO from NO_2_ [133,134]. A distinct property between the two gases is that NO is a colorless gas while NO_2_ is reddish-brown in color [135]. Due to its reduced electron valence in NO, first-principle studies demonstrated that the adsorption energy of NO to be slightly higher than that of NO_2_ [136]. Nevertheless, the binding of NO with MoS_2_ is still considered one of the strongest in many gas molecules including CO, CO_2_, NH_3_, CH_4_, H_2_O, N_2_, O_2_, and SO_2_ [136]. This computational finding was verified by a few experiments that successfully detected NO with MoS_2_ materials down to the ppm level [137,138,139]. Although there is not much research work that focuses on NO detection, it is demonstrated to give promising performances using TMDC material (see Table 2).

### 3.2. Ammonia Detection

**Figure 3:**
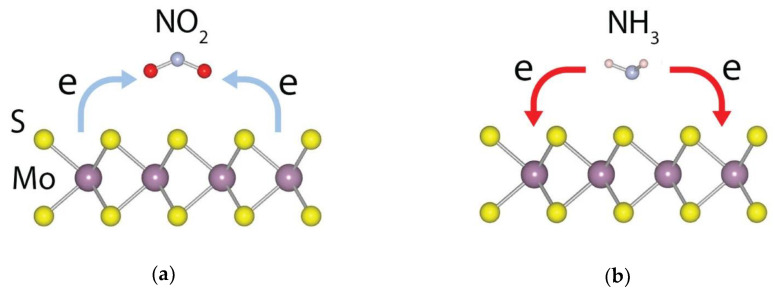
(**a**) Mechanism of electron-accepting NO_2_ gas molecule on MoS_2_ surface and (**b**) mechanism of electron-donating NH_3_ gas molecule on MoS_2_ surface (Reproduced with permission from [113]. Copyright 2015 Springer Nature Limited CC BY-NC-ND 4.0).

Ammonia is an indirect greenhouse gas, as it is quickly soluble by water vapor or rain in the atmosphere and goes down to the land to be readily used as fertilizers by plants [141]. The lifespan of this alkaline and reactive gas in the atmosphere is short, around 1 day [142]. Nevertheless, direct exposure to ammonia gas from its emission source can still cause health issues to humans [143]. Gases such as NO_2_ are electron-accepting, while NH_3_ donates electrons to the TMDCs surface [113] to give a change of resistivity in the material (see Figure 3a,b). Due to their different adsorption mechanism, NO_2_ and NH_3_ are two flagship gases to study the generic effect of gas sensing [113,144,145].

Many works have also indicated that TMDC materials have excellent properties to act as excellent ammonia gas sensors [145]. For example, Cho et al. [146] used a 2D graphene/MoS_2_ heterogeneous structure to improve the sensitivity of the sensor towards gas molecules. Burman et al. [139] studied the effect of vacancy sites on MoS_2_ sensors and proposed a UV-treated sensor that can be synthesized from powder. Other TMDC materials such as MoTe_2_ [147] and WS_2_ [148] also showed performances of being able to detect ammonia gases down to ppm levels. Even artificial neural networks were used to post-process signals from TMDC ammonia gas sensors to improve gas concentration predictions [144]. A few of the significant works of applying TMDC materials as ammonia gas sensors are found in Table 3.

### 3.3. Volatile Organic Compound (VOC) Detection

Volatile organic compounds (VOC), commonly known as aromatic hydrocarbons fractions, are organic chemicals that have high vapor pressure in atmospheric conditions. VOC are studied to be emitted in landfills [153], newly renovated buildings [154], industrial refineries [155], and other leakage sources. Long-term exposure to VOC can lead to dysfunction of central nervous systems, memory loss, and cause congenital anomalies for reproduction [156]. VOC sensors are important for environmental detection [154], industrial emission control [157], and even for cancer diagnosis [158].

**Figure 4:**
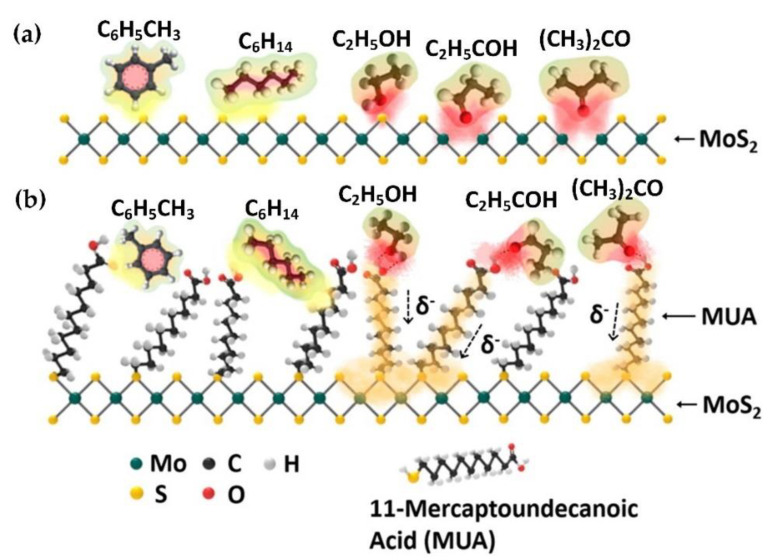
(**a**) Gas sensing mechanism for volatile organic compounds (VOC); toluene, hexane, ethanol, propanal, and acetone) gas molecule on primitive MoS_2_ surface and (**b**) improved VOC sensing performance by Mercaptoundecanoic acid (MUA)-conjugated MoS_2_ due to increased charge carrier density (Reproduced with permission from [160]. Copyright 2014 American Chemical Society).

The challenge of designing sensors for VOC is that VOC is not a single gas molecule but can consist of multiple molecules (such as benzene, ethylene glycol, formaldehyde, etc.) with slightly different properties. Barzegar et al. [159] represented the VOC molecule behavior by using xylene isomers and methanol, showing high potential for adsorption between VOC molecules and a Ni-decorated MoS_2_ sensor. A thiolated ligand conjugate MoS_2_ sensor was also shown to exhibit excellent sensitivity down to a concentration of 1 ppm [160]. The work represented VOC by studying a combination of toluene, hexane, ethanol, propanal, and acetone gas molecules while demonstrating the conjugation of thiolated ligand improves the charge carrier density of the sensor (see Figure 4). A recent work from Tomer et al. [161] demonstrated that a cubic Ag(0)-MoS_2_ loaded g-CN 3D porous hybrid had multifunctional abilities towards sensing VOC by studying *n*-butanol, isopropanol, benzene, and xylene gas molecules. Short response time and recovery time of 7–15 s and 6–9.5 s respectively were obtained for all VOC molecules at 175 °C. Zhao et al. [162] presented an optical VOC microsensor using a photonic crystal cavity integrated with MoS_2_. VOC molecules of acetone, methanol, ester, dichloromethane, and methylbenzene were studied and the optical microsensor resulted in a LOD of 2.7 ppm, response time of 0.3 s, and recovery time of 100 s.

### 3.4. Detection of Sulphur Gases and Other Gases

The unique properties of TMDC materials also showed many preliminary potentials for the detection of sulphur gases and other emissions. Chen et al. [163] demonstrated from density functional theory (DFT) that MoS_2_ monolayers exhibit great adsorption energy towards SF_6_ decomposition (which includes SO_2_, SOF_2_, SO_2_F_2_, H_2_S, and HF). The adsorption of SF_6_ decomposition by monolayer PtSe_2_ was also studied by DFT methods and found to give excellent adsorption energies [164]. Park et al. [165] experimentally demonstrated that a Pt nanoparticle decorated MoS_2_ gas sensor can achieve high sensitivity detection of H_2_S down to 30 ppm. Recent work from Yang et al. [166] demonstrated that Ni- and Cu-embedded MoS_2_ monolayers can improve the adsorption energy of SO*_x_* and O_3_ molecules compared to that of the pristine MoS_2_.

**Figure 5:**
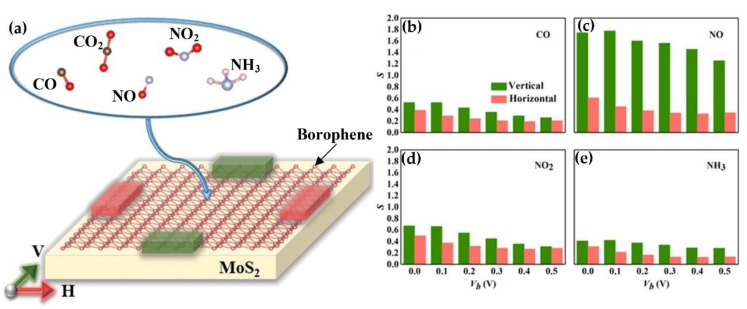
(**a**) Direction-sensitive borophene/MoS_2_ heterostructure for multiple climate gas detection. Sensitivity and directional bias voltage of (**b**) CO, (**c**) NO, (**d**) NO_2_, and (**e**) NH_3_ in the horizontal and vertical direction (Reproduced with permission from [168]. Copyright 2020 Elsevier B.V.).

TMDCs also demonstrate adsorption and sensing ability for CO gas. The work of Ma et al. [167] studied the potential of sensing CO and NO gas molecules by doping metal particles on MoS_2_ monolayer. The doping of Au, Pt, Pd, and Ni has shown to alter the transport property of MoS_2_, giving better adsorption energy for CO and NO gas detection. Recently, Yang et al. [91] presented a strategy of using Ti doping and the application of an electric field to improve adsorption energies in MoSe_2_ and MoS_2_ materials. The work concluded that Ti doping strategy improved the sensitivity of both materials towards CO and NO gas detection. Another recent work from Shen et al. [168] provided a study of using a borophene/MoS_2_ heterostructure to detect small gas molecules (CO, NO, NO_2_, and NH_3_), which exhibited different resistive properties towards these molecules when changing voltage direction (see Figure 5). Modulation of MoS_2_ by antisite doping and strain also showed improved gas detection sensitivity [169]. Ma et al. [170] studied the effects of defects on WSe_2_ monolayer sensor and found that Se vacancies can improve sensing ability of H_2_O and N_2_ molecules. Further interest gas detection also extends towards Cl_2_, PH_3_, AsH_3_, BBr_3_, and SF_4_ gas molecules on MoS_2_ [171]. More materials will be expected to be discovered for gas detection applications in the future.

## 4. TMDC Materials for Wastewater Treatment

The United Nations has highlighted that 80% of wastewater is released back to the environment without sufficient treatment [172]. The effect of industrial development and human activities has released many pollutants into the water. The major water pollution sources come from various source such as industry waste [173], sewage and wastewater [174], oil leakage [175], chemical fertilizer and pesticides [176], and mining activities [177]. These pollutants require high oxygen for oxidation decomposition, which reduces the dissolved oxygen level in the water that will damage the aquatic ecosystem [178]. Wang et al. [179] reviewed that many technologies including adsorption, ion exchange, membrane filtration, chemical precipitation, and electrochemistry have been widely used in water treatment. Despite the application of various technologies, quality water supply remains unsustainable [180]. Many researchers have reported promising outcomes with the application of nanomaterial such as TMDC-based material in water treatment technology [181].

### 4.1. Adsorption for Wastewater Treatment

**Figure 6:**
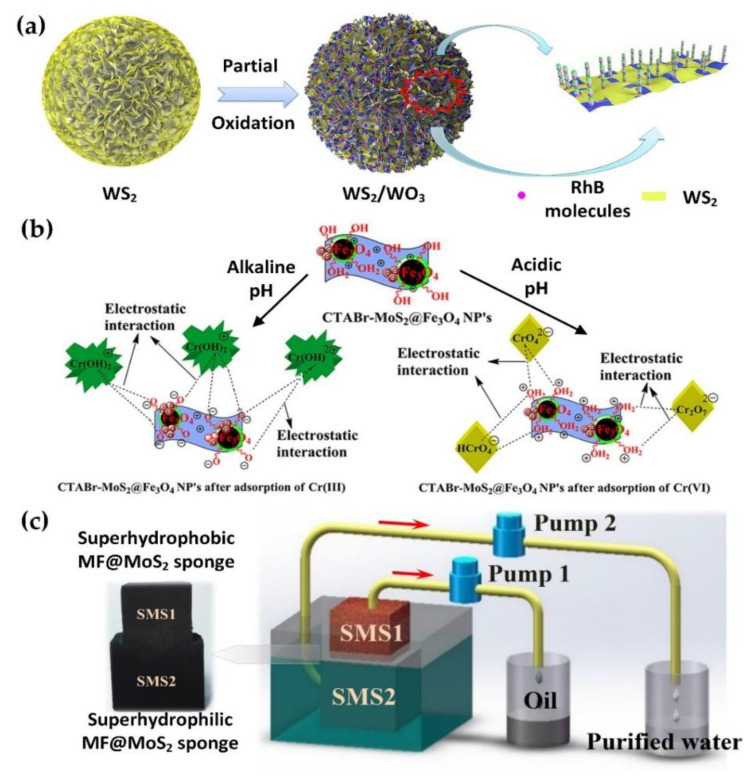
(**a**) Facile oxidation strategy to prepare a flower-like WS_2_/WO_3_ heterostructure for adsorbing RhB from wastewater (Reproduced with permission from [182]. Copyright 2020 MDPI AG CC-BY 4.0). (**b**) Bifunctional cetyltrimethylammonium bromide (CTABr)-coated MoS_2_-decorated Fe_3_O_4_ nanoparticles using pH to alter adsorption properties between Cr(VII) and Cr(III) in wastewater (Reproduced with permission from [189]. Copyright 2017 American Chemical Society CC-BY-NC-ND Under ACS AuthorChoice). (**c**) A superhydrophobic/superhydrophilic MF@MoS_2_ sponge for water/oil separation (Reproduced with permission from [190]. Copyright 2017 Elsevier B.V).

Among wastewater treatment technologies, the adsorption is considered the most inexpensive, fast and simple operation method [182]. Recently, researchers are focusing on the application of TMDC-based material in adsorption technology. That MoS_2_ nanosheet is used in the adsorption process mainly due to its large surface area, excellent chemical and thermal stability and environmentally friendly [183]. Many application of TMDC-based material has been successfully demonstrated with an effective outcome including the removal of methyl orange [184], Rhodamine B (RhB) [185], and Congo Red [186]. Li et al. [187] stated that the application of MoS_2_ adsorbent can increase the adsorption capacity in removing organic dye. An alternative facile oxidation strategy was proposed by Li et al. [182] to synthesize tungsten disulphide/tungsten trioxide (WS_2_/WO_3_) heterostructures to remove RhB molecules from wastewater (see Figure 6a). This strategy has contributed to higher adsorption capacity through manipulating the surface property. Massey et al. [188] further added that the hierarchical microsphere of MoS_2_ nanosheets has demonstrated high adsorption capacity with 297, 216, and 183 mg/g of methylene blue, rhodamine 6G, and fuchsin acid dye respectively as compare to conventional absorbent such as activated carbon. Most importantly, the hierarchical microsphere of MoS_2_ nanosheets can be regenerated effectively without affecting the performance of adsorption capacity.

Kumar et al. [189] concluded that the synthesis of MoS_2_ with magnetic nanoparticles can be effective adsorption material especially in removing heavy metal such as chromium. The efficiency of chromium removal is highly dependent on the pH of the solution and the adsorption of Cr(VII) and Cr(III) can be selected by changing the pH (see Figure 6b). Besides that, the regeneration of absorbent does not reduce the efficiency of chromium uptake significantly.

On top of that, the CeO_2_-MoS_2_ hybrid magnetic biochar (CMMB) also exhibits a strong magnetic ability to remove lead(II) (Pb(II)) and humate from water treatment [191]. The CMMB can remove >99% of Pb(II) and humate within 6 h. The application of MoS_2_/thiol-functionalized multiwalled carbon nanotube (SH-MWCNT) has also displayed high adsorption capacity for heavy metal removal [177]. However, the spent adsorbent can be further used for photocatalytic and electrochemical applications.

Furthermore, the synthesized MoS_2_-coated melamine-formaldehyde (MF@MoS_2_) sponges that exhibit a superhydrophobic and superhydrophilic characteristic (see Figure 6c) demonstrated a highly selective adsorption capacity [190]. The MF@MoS_2_ sponges have high absorption performance for oil and organic solvent and water-soluble dye. It also exhibits high discoloration efficiency of 98% methyl orange within 10 min. Wan et al. [192] added that the MF@MoS_2_ can be modified from room temperature vulcanized silicon rubber.

### 4.2. Membrane Technology in Wastewater Treatment

In water treatment systems, the typical microporous membrane pore size is about 0.1–5 µm, which limits membrane technology on water purification [179]. The development of nanoporous membranes exhibited high filtering performance in dealing with most of the pollutants inducing microbes, organic molecules, heavy metal, and salts.

**Figure 7:**
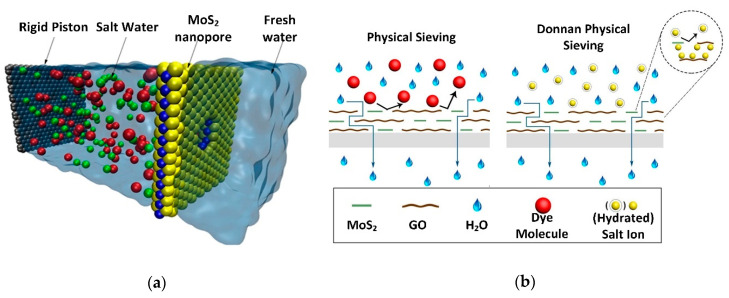
(**a**) Single-layer MoS_2_ water desalination membrane with Mo only pores (Reproduced with permission from [193]. Copyright 2015 Springer Nature Limited CC BY 4.0). (**b**) MoS_2_/GO nanosheets intercalated membrane for removal of dyes and salts (Reproduced with permission from [196]. Copyright 2020 Elsevier B.V.).

Heiranian et al. [193] highlighted that the single-layer MoS_2_ membrane with a full Mo pore (see Figure 7a) exhibited 88% of ion rejection. The work also demonstrated that the water flux was two to five orders of magnitude greater than other known nanoporous membranes. This technology is crucial to replacing the reverse osmosis (RO) membrane, especially in water desalination processes. Later, Kou et al. [194] discussed that a 0.74 nm nanopores in monolayer MoS_2_ membranes should be compatible with Debye screening length for the electrostatic interaction and lesser than the mean free path of molecules in water. Recent work from Kozubek et al. [195] proposed using irradiation with highly charged ions (HCIs) to create pores in MoS_2_. They found that pore creation efficiency has a linear relationship with potential energies for pore radius of 0.55–2.65 nm. Kozubek et al. [195] added that the HCI method has parallel writing capabilities, giving a high potential for mass production.

In the application of water desalination, Ma et al. [196] stated that the synthesis of MoS_2_/GO membrane resulted in an enhanced water flux (from 8.83 to 48.27 L·m^−2^∙h^−1^) with improved salts removal capacity (from 54.32% to 96.85%). The MoS_2_/GO membrane also exhibited different mechanism for dyes and ions (see Figure 7b) where dyes are physically sieved while ions have a Donnan effect due to existing trapped ions within interlayer spacing [196]. Gao et al. [197] demonstrated the performance of MoS_2_ membrane’s performance in rejecting 100% of methylene blue dye. The work highlighted that the increase in nanosheets to the membrane will reduce permeability and increase dye rejection rate.

### 4.3. Photocatalyst Technology in Wastewater Treatment

**Figure 8:**
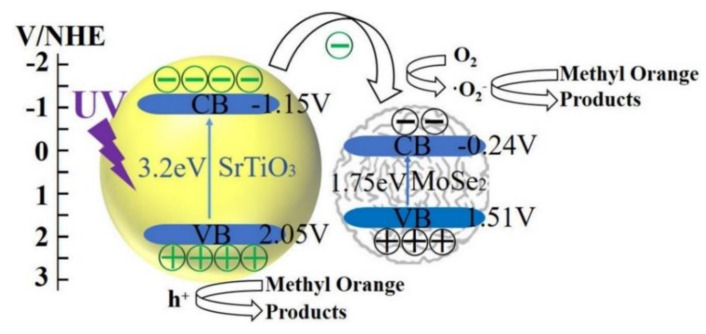
Mechanism of a MoSe_2_/SrTiO_3_ photocatalyst to oxidize and degrade methyl orange dyes in wastewater. UV irradiation causes electron transfer from the valence band (VB) to the conduction band (CB), which activates the separation process (Reproduced with permission from [200]. Copyright 2018 World Scientific Publishing Co Pte Ltd CC BY Under Open Access).

As the majority of water treatment facilities are located outdoors, the availability of solar energy can be useful for photocatalytic technology in water treatment. Chu et al. [198] reported that the development of hexagonal 2H-MoSe_2_ photocatalyst exhibits outstanding photo-absorption and photocatalytic reaction in reducing hexavalent chromium (Cr(VI)) under ultraviolet (UV) light. The 2H-MoSe_2_ yields 99% Cr(VI) reduction rate with the presence of UV light. Mittal and Khanuja [199] revealed that MoSe_2_ is a good photocatalyst not only for Cr(VI) but also for methylene blue (MB) and RhB. The MoSe_2_/strontium titanate (SrTiO_3_) heterostructure also showed great potential as a wastewater treatment photocatalyst with a degradation rate of methyl orange at 99.46% under the optimum loading weight of 0.1 wt.% under UV light [200]. It is also found that there is no significant loss in the performance of MoSe_2_/SrTiO_3_ after reuse for 6 times. The working principle of MoSe_2_/SrTiO_3_ photocatalyst is demonstrated in Figure 8.

## 5. Fuel Cleaning

Fuels are means of transfer for energy in our ecosystems. Most fuels have many imperfections related to conversion inefficiencies [201] and fuel impurity (including sulphur, nitrogen, aromatic, etc.) [202]. Direct usage of crude fuels with such impurity content will lead to an increase in gas emissions [203,204]. Most refineries for oil-based fuel carry out fuel cleaning processes such as desulphurization, dearomatization, denitrogenation, and deoxygenation [202,205,206] to remove such impurities before the fuel is being converted. The use of impurity removal technology generally depends on the techno-economics and fuel costs during application [207]). Fuels with a high impurity such as sulphur content can potentially cause an elevated particulate emission [208] while causing catalyst poisoning in subsequent systems [209].

### 5.1. Fuel Hydrodesulfurization

Hydrodesulfurization is the process of reduction of sulphur content from fuel such as diesel [202] by catalytically reacting them with hydrogen. Paul et al. [210] studied the mechanism of vacancy formation on the MoS_2_ catalyst in the hydrodesulfurization process, demonstrating the activation energy of 0.5 eV. TMDC materials such as Co/Ni promoted MoS_2_ were popular for the use of hydrodesulfurization due to its high activity, good selectivity, resistance to deactivation, and regeneration ability [211]. Commonly, TMDC-based catalysts are used for hydrodesulfurization in the oil and gas industry for high-efficiency sulphur removal from naphtha [212]. For the hydrodesulfurization of naphtha, a recent work from Mahmoudabadi et al. [213] achieved 100% conversion efficiency using MoS_2_ quantum dots nanocatalyst under the pressure of 15 bars, temperature of 280 °C and liquid hourly space velocity (LHSV) of 4 h^−1^. The direct use of unsupported Ni/MoS_2_ catalyst was reported [214] to remove furfurylamine (FA) and dibenzothiophene (DBT). Liu et al. [215] demonstrated that for a MoS_2_/NiMo catalyst, the higher the number of MoS_2_ slabs being stacked, the better the hydrodesulfurization selectivity for DBT. Additionally, Rangarajan et al. [216] studied the preferred active sites of a metal-promoted MoS_2_ and found that organosulfur and organonitrogen compounds bind weaker on sites with exposed metal on the corner and the sulphur edge of MoS_2_. Hydrodesulfurization of DBT by various morphologies of MoS_2_ catalyst was also studied by Tye and Smith [217]. This work showed that exfoliated MoS_2_ has the highest conversion, compared to the commercially crystalline MoS_2_ powder and MoS_2_ derived from soluble Mo precursors. While selectivity was correlated to the edge sites of MoS_2_. A 3D NiS-MoS_2_/Graphene nanohybrid was also shown to give a high DBT conversion rate of 82.6%, showing potential for 3D composites. Recent work from Abbasi et al. [218] demonstrated that a cobalt-promoted MoS_2_ achieved more than 80% conversion for the application of diesel, showing potential for the transfer of high-performance TMDC catalysts from oil and gas to diesel/biodiesel industries.

Besides hydrodesulfurization to remove DBT, the removal of thiophene was also demonstrated to be feasible with MoS_2_ catalysis by DFT methods [219]. Kaluza et al. [220] synthesized a MoS_2_ catalyst supported on mesoporous alumina that gave a thiophene conversion of more than 60% at around 400 °C. For the removal of carbonyl sulphide (COS), Liu et al. [221] used a microwave activated MoS_2_/graphene catalyst that achieved over 90% conversion for temperatures over 280 °C. Later, the use of a monolayer MoS_2_ anchored on reduced graphene oxides [222] has shown to give over 90% conversion and a reduced temperature at about 180 °C.

### 5.2. Fuel Hydrodeoxygenation

**Figure 9:**
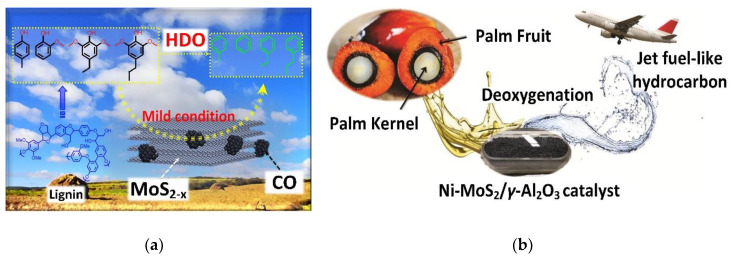
(**a**) Hydrodeoxygenation (HDO) of lignin-derived phenols into arenes using Co-MoS_2−*x*_ (Reproduced with permission from [225]. Copyright 2020 American Chemical Society) and (**b**) deoxygenation of palm kernel oil to jet fuel-like hydrocarbon using catalysts (Reproduced with permission [206]. Copyright 2017 Elsevier B.V.).

Hydrodeoxygenation is the removal of oxygenated components from the fuel by reacting it with hydrogen to form water [206]. Liu et al. [223] found that a MoS_2_ monolayer doped with an isolated Co atom can effectively reduce hydrodeoxygenation of 4-methylphenol to toluene from 300 °C to 180 °C while maintaining high conversion and selectivity of 97.6% and 98.4% respectively. From a DFT perspective, Li et al. [224] demonstrate a generic structure of Co atom vacancy in MoS_2_, claiming that the catalyst can activate hydrodeoxygenation reactions at lowered temperatures. Recent work from Wu et al. [225] adsorbed Co oxide at the edge of MoS_2_ with sulphur defects and formed a Co-MoS_2−*x*_ catalyst that converts lignin-derived phenols (4-methylphenol) to arenes with 97.4% conversion and 99.6% toluene selectivity at a mild temperature of 120 °C (see Figure 9a). For direct industrial application, palm kernel oil was converted with a yield over 90% to jet fuel-like hydrocarbon (see Figure 9b) via a Ni-MoS_2_/*γ*-Al_2_O_3_ hydrodeoxygenation catalyst [206]. The work showed high potential for converting biomass-based fuel to high-value fuels using TMDC-based catalysts. Moreover, Co- and Ni- promoted MoS_2_ catalyst was also used to co-process diesel and vegetable oil via hydrodeoxygenation reaction [226]. Alvarez-Galvan et al. [227] demonstrated that transition metals phosphides were useful as a hydrodeoxygenation catalyst for waste cooking oil to green biodiesel. More research work is required to explore the possibility of TMDC materials in this field.

For catalytic deoxygenation of alkali lignin into bio-oil, Li et al. [228] proposed the use of a flower-like hierarchical MoS_2_-based composite catalyst that achieved a lignin conversion of 91.26% and bio-oil yield of 86.24%. Later, work from Zhou et al. [229] synthesized a series of MoSe_2_ catalysts for the conversion of alkali lignin into bio-oil. The work resulted in 96.46% conversion of lignin and 93.68% yield of bio-oil. A recent review by Porsin et al. [230] revealed that sulphide catalysts such as MoS_2_ can also convert fatty acid triglycerides to motor oil by hydrodeoxygenation. As for wider fuel cleaning applications, TMDC materials have also been reported to purify gas fuels such as hydrogen and methane [231] showing high potential in diverse fuel applications in the future.

## 6. CO_2_ Valorization and Conversion

**Figure 10:**
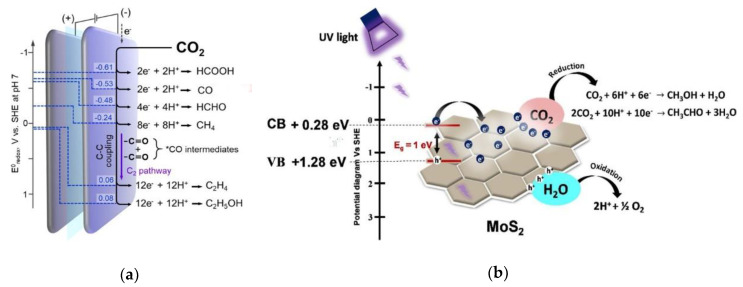
(**a**) Scheme of the electrochemical conversion of CO_2_ into value-added chemicals (Reproduced with permission from [241]. Copyright 2019 CC BY-NC 4.0 John Wiley and Son) and (**b**) general working principle of photocatalyst for CO_2_ conversion (Reproduced with permission from [249]. Copyright Elsevier 2019).

Valorization of carbon dioxide (CO_2_) to raw chemical materials and clean fuels is an opportunity for the artificial carbon cycle, which contributes to the mitigation of global warming and alleviates the usage of fossil [232]. Over the decades, enormous efforts in searching of alternative technologies to mitigate the CO_2_ emissions through CO_2_ capture from concentrated industrial exhausts [233,234], sequestration of CO_2_ in the underground [235,236] and conversion of CO_2_ to energy-rich fuels powered by renewable energy resources [237] have been discovered. Throughout all these methods, CO_2_ molecules are not solely can be removed from the atmosphere but also can be converted into value-added chemicals such as methanol, formic acid, methane, and syngas [238,239,240]. Recently, electrochemical conversion of CO_2_ to value-added chemicals through the principal of CO_2_ electrochemical reduction reaction (CO_2_ERR) as shown in Figure 10a [241], has emerged as a comparative alternative to its counterparts such as biochemical and thermochemical technologies [242,243,244]. Alternatively, semiconductor-based photocatalysis of CO_2_ reduction has been another frontier in CO_2_ conversion [245]. When a light source with appropriate photoenergy is illuminated on this photocatalyst, electron-hole pairs are formed [246,247]. With the migration of the electron-hole pair to the surface, the release of energy from within can reduce surface adsorbed CO_2_ [246]. Another interesting type of photocatalyst is the Z-scheme photocatalyst, which performs charge transfer between a reduction semiconductor catalyst and an oxidation semiconductor catalyst, potentially giving higher performances, efficiency, and synthesis possibilities [248]. The general working principle of photocatalyst for CO_2_ conversion is illustrated in Figure 10b.

### 6.1. Conversion of CO_2_ to Syngas and Other Gases

To achieve this attractive blueprint, the key issue is to develop electrocatalysts with high activity, superior selectivity, highly durable, environmentally friendly as well as low cost [250,251,252]. In 2014, the first pioneering work of elucidating the TMDCs materials as advanced electrocatalysts for CO_2_ reduction was by Asadi and coauthors [253]. They have claimed that MoS_2_ material serves as a highly efficient electrocatalyst for the conversion of CO_2_ to syngas. The combination of the edge states of MoS_2_ in contact with ionic liquid solvent electrolytes has served as a new paradigm for CO_2_ reduction, providing the advantage of favorable electronic properties of MoS_2_ and an electrolyte that transfer the CO_2_ molecules to the active sites for reaction. This study is a breakthrough in this field, in which the performance of the catalytic activity for CO_2_ reduction reaction is far exceeding than other conventional catalysts such as carbon nanotubes, graphene, noble metal carbides, and transition metals.

**Figure 11:**
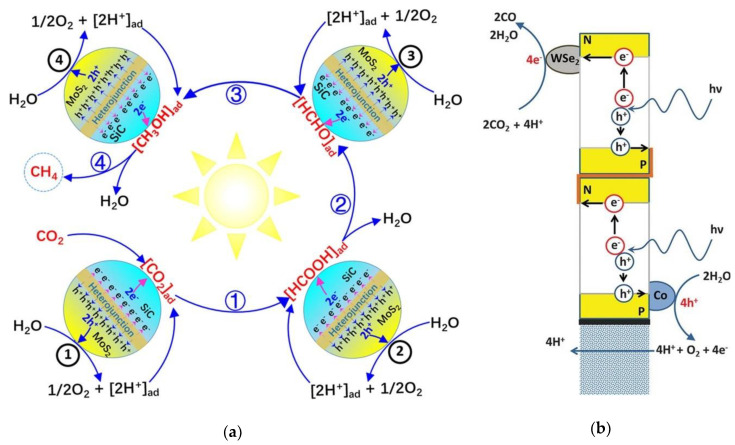
(**a**) Photochemical reaction pathway for the conversion of CO_2_ and H_2_O to CH_4_ and O_2_ under visible light using SiC@MoS_2_ nanoflower (Reproduced with permission from [254]. Copyright 2018 American Chemical Society) and (**b**) schematic of the WSe_2_-based artificial leaf cell design for the conversion of CO_2_ and H_2_O to CO and H_2_ (Reproduced with permission from [255]. Copyright 2016 The American Association for the Advancement of Science).

Recent research work of TMDCs in converting CO_2_ to interesting gas products using photo- and electro-catalysis methods are shown in Table 4. Up to date, most reports of TMDCs in this field are focusing on the conversion of CO_2_ to CO, suggesting that there is still a huge potential of different electrochemical reaction pathways using TDMCs that can be explored in the near future such as the conversion of CO_2_ to CO and H_2_ to CH_4_. Wang et al. [254] synthesized a marigold-like SiC@MoS_2_ nanoflower for the conversion of CO_2_ and H_2_O to CH_4_ and O_2_ using no sacrificial agents while operating within the visible light spectrum (see Figure 11a). The work reported production rates of 323 and 621 µL∙g^–1^∙h^–1^ for CH_4_ and O_2_ while maintaining stable characteristics for 40 h. Asadi et al. [255] also have synthesized a series of TMDCs (e.g. WSe_2_, WS_2_, MoSe_2_, and MoS_2_) using the chemical vapor transport (CVT) growth technique followed by liquid exfoliation for electrochemical CO_2_ conversion using ionic liquid electrolyte (EMIM-BF4). By benchmarking with the bulk Ag and Ag NPs, the current density of TMDCs are more than tenfold (130–330 mA cm^−2^) of the current density of Ag (3.3 mA cm^−2^) and Ag NPs (10 mA cm^−2^) with 90% CO. Overall, WSe_2_ NFs gave the best performance and exhibited a current density of 18.95 mA cm^−2^ with a high turn-over frequency of CO of 0.28 s^−1^ at an overpotential of 54 mV. This can be related to the intrinsic properties of WSe_2_ (very low work function and high-volume surface area).

In addition, a lab-scale synthetic custom-builds wireless setup for photochemical studies of WSe_2_/IL is also being developed by this group [255]. The build-up of the artificial leaf that performed the photosynthesis process is investigated to further confirm the CO_2_ conversion rate efficiency (see Figure 11b). The system comprises of three main components: (i) harvest light using two amorphous silicon triple-junction photovoltaic cells in series, (ii) CO_2_ reduction using WSe_2_/IL on the cathode and lastly, and (iii) evolution of oxygen using cobalt (CoII) oxide/hydroxide in potassium phosphate electrolyte [265,266]. Surprisingly, the cell managed to function continuously for more than 4 h before corrosion happens at the transparent indium tin oxide layer on the anode, proving that this theoretical experiment is workable and insightful, which could be extended to the technology development scale in near future. Further studies on the defect engineering for CO_2_ reduction catalysts were also discussed by Wang et al. [267] to improve functionality by defect engineering (such as holes, doping, vacancies, phase change, edge, grain boundary, lattices distortion, and other methods). For this, MoS_2_ with substitutional defects on the edge was studied [253] and showed to give better current density than Au, Ag, Cu nanopowder, and the polycrystalline catalyst. Ji et al. [268] suggested that group 10 TMDCs have a high potential for CO_2_ reduction as they do not suffer from OH poisoning. The work proposed that the reaction energies for CO_2_ reduction could be tuned by vacancy densities.

### 6.2. Direct Conversion of CO_2_ to High-Value Chemicals

Direct conversion of CO_2_ to high-value chemicals (such as intermediate chemicals, fuels, and alcohols) has a high potential for commercial value [269,270]. TMDCs have demonstrated properties for catalyzing CO_2_ into important alcohol, especially methanol [85,271]. Bolivar et al. [272] studied the use of a Pt/MoO_x_/MoSe_2_ electrocatalyst and proposed that oxygen-containing surface species lowers the electric potential for methanol production. Moreover, Tu et al. [273] have demonstrated the methanol production of TiO_2_ and 0.5 wt.% MoS_2_ nanocomposites was higher than Pt/TiO_2_, Au/TiO_2_, and Ag/TiO_2_ for the photocatalytic reduction of CO_2_. For this TiO_2_/MoS_2_ composites, Xu et al. [274] showed that electrons are transferred from MoS_2_ to TiO_2_ upon contact, therefore promoting the separation of charge carriers upon photoexcitation. In 2018, Francis et al. [275] have reported on the activity of single crystals and thin films of MoS_2_ for the reduction of CO_2_ dissolved in an aqueous electrolyte to yield 1-propanol, ethylene glycol, and t-butanol and hydrogen. At an applied potential of −0.59 V, the Faradaic efficiencies for the reduction of CO_2_ to 1-propanol are *ca.* 3.5% for MoS_2_ single crystals and 1% for thin films with low edge-site densities. WS_2_ quantum dots doped Bi_2_S_3_ nanotubes also demonstrated high (38.2 μmol∙g^−1^∙h^−1^) production of methanol while co-producing ethanol [85]. Many researchers have also performed the conversion of CO_2_ to high-value products such as methanol, acetaldehyde [249] and ethanol [276] by using TMDC catalysts (see Table 5).

## 7. Challenges and Future Prospects

The application of TMDCs in pollution reduction is a breakthrough as has high availability [30,31,32], high economic potential [280,281,282], and excellent efficiency [254,276] for the application of pollution reduction. For TMDCs availability and efficiency, we have already discussed them in Section 1 and Section 2, Section 3, Section 4, Section 5 and Section 6 (depending on application) respectively. In terms of commercial techno-economic analysis, a few works have also pointed out the advantage of using TMDC catalysts. Valle et al. [280] studied the economics of converting biomass-derived syngas to ethanol using an indirect circulating fluidized bed gasification (iCFBG) system via the KCoMoS_2_ catalyst. The work highlighted that the iCFBG system could reach a minimum ethanol selling price (MESP) of 0.74 $/L (USD) while technologies such as entrained-flow gasification have that at 0.98$/L. This MoS_2_-based catalyst was also found to be more commercially viable than the Rh-Mn/SiO_2_ catalyst due to their cheaper economical costs, although the latter has better energy efficiency [281]. Phillips [282] also concluded that the use of Co/MoS_2_ has potential to lower down ethanol MESP down to 0.267 $/L (USD) highlighting that Ru or Rh-based catalysts are economically expensive even at low concentrations. Nevertheless, authors acknowledge that the techno-economic analysis of cutting-edge TMDCs technologies is still generally unknown. Thus, more TMDCs research studies should include consideration of economic analysis. Furthermore, despite the unique physiochemical properties offered by the TMDCs (e.g., the large surface area, which acts as a two-edged sword [283], tunable absorption heterostructure [200,284], flexible, diversiform, and functional properties [146]), more exploration on the operating conditions, kinetic, and thermodynamic parameters should be carried out to further confirm its ability [285]. This is because most of the reviewed works are conducted under a controlled environment with a specially designed flow. Under this context, whether obtained simulation and experimental results reflect the actual situation remains uncertain. The main limitations of commercializing of TMDCs-based technology are:

(i) Most research works are focusing on the development of the materials as an effective pollutant degradation catalyst during oxidation, neglecting that in reality, the number of organic pollutants is in a mixture that may affect the performance of the engineered material [286,287].
(ii) The rate of product formation for TMDC-based technology, such as syngas and methanol from the conversion of CO_2_ is still far from the industrial scale, which is not sufficient to accommodate the global demand [286,287]. More research effort is required to improve efficiencies.
(iii) High preparation/fabrication cost and poor uniformity of the materials in large-scale production [288]. More effort is required in sustainable synthesis and fabrication of TMDC-based technologies.
(iv) The larger surface area offered by the TMDCs can enhance the adsorption capability, but also cause higher effects of environmental disturbance [283]. Precise defect engineering to improve material performances can also be carried out [225,267]. Optimization and data analytics on the design dilemma and defect engineering should be highlighted and properly studied.

As a future prospect, commercial validation on the performance on a larger scale needs to be carried out. The results obtained from the experiment can be validated by conducting techno-economic analysis, such as Monte Carlo analysis for economic and risk feasibility [289,290]. Furthermore, advanced machine learning methods can also be used to accelerate experimental designs [291,292], obtain optimal performances [293,294], and for the discovery of new TMDCs composite structures [295,296].

## 8. Conclusions

The unique and exclusive features of TMDCs (e.g., layered structure, tunable bandgap, unique optical, thermal and electrical properties, etc.) have been the main driving force that drives the researchers’ attention on exploring the potential of the 2D materials in pollution reduction applications. This work summarized the state-of-the-art applications of various TMDCs under the context of pollution mitigation (including (i) gas adsorption and removal, (ii) gas sensing, (iii) wastewater treatment, (iv) flue cleaning, and (v) CO_2_ valorization and conversion). In addition to the up-to-date progress of TMDCs research, this article also discussed some of the key challenges for the future commercialization of TMDC materials. Apparently, many of the reviewed research works have authenticated their substantial potential to substitute the existing pollution mitigation media. Nevertheless, the current applications are still restricted to a lab basis, where the deviation of the actual performance of TMDCs under larger-scale production remains as the research gap. To usher TMDCs into the next level of utilization (i.e., from the lab scale to the industrial scale), the following three research directions should be followed up, (i) techno-economic analysis (TEA) study, (ii) experimentation under more rigorous and realistic conditions, and (iii) experimental optimization for application purpose. The authors sincerely hope this review can serve as an insightful guideline that inspires more researchers to venture into this new and exciting cutting-edge field.

## Figures and Tables

**Table 1:** NO_2_ sensing TMDC materials and their performances.

Material	Target Gas	LOD	Condition	Remarks
MoS_2_/graphene hybrid aerogel [120]	NO_2_	50 ppb	RT to 200 °C	Response and recovery time < 1 min.
WS_2_ with AgNW functionalization [128]	NO_2_	25 ppm	100 °C	667% response compared to pristine 4L WS_2_ sensor.
MoS_2_ [121]	NO_2_	20 ppb	RT	Sensitivity of 194%/ppm
MoS_2_ [129]	NO_2_	120 ppb	RT, 100 °C	Peak to valley sensitivity > 50%.
MoS_2_ [115]	NO_2_	25 ppb	RT (with red light)	Sensitivity of 3300%/ppm.
rGO/MoS_2_ nanocomposite [123]	NO_2_	5.7 ppb	60 °C	Over 55% sensing response for NO_2_ at 8 ppm
ZnO/MoS_2_ [130]	NO_2_	200 ppb	RT (with monochromatic light)	Sensitivity at 29.3%/ppm, response time of 4.3 min, recovery time of 1.2 min.
SnS_2_/MoS_2_ [114]	NO_2_	25.9 ppm	RT	Response time of 2 s and recovery time of 28.2 s.
MoSe_2_ [131]	NO_2_	300 ppm	RT	Response time of 20 min and recovery time of 30 min.
MoSe_2_ [116]	NO_2_	10 ppm	RT (with UV light)	Response time <200 s.
WS_2/_WO_3_ composite film [132]	NO_2_	100 ppb	150 °C	Response time of 70 s and recover time of 120 s.
WS_2_/graphene aerogel [122]	NO_2_	10-15 ppb	RT	Response time of 70 s, recovery time of 300 s. (2 ppm)
MoTe_2_ [127]	NO_2_	20 ppb	RT (with UV of 2.5 mW/cm^2^)	Response time of 120 s with response of 18%.
PtSe_2_ [126]	NO_2_	0.9 ppb	RT	Response time of 1 s and recovery time of 4 s

**Table 2:** NO sensing TMDC materials and their performances.

Material	Target Gas	LOD	Condition	Remarks
MoS_2_ deposited onto Si/SiO_2_ [137]	NO	0.8 ppm	RT	80% decreased response at 2 ppm.
MoS_2_ [138]	NO	100 ppm	RT, 50 °C, 100 °C (with UV light)	Response and recovery time below 600 s. Response at 25.63%.
UV-ozone treated MoS_2_ [139]	NO	20 ppm	125 °C	Stability issue over 120 ppm. Poor recovery.
WO*_x_*/WSe_2_ hybrid [140]	NO	0.3 ppb	RT	Response time of 250 s, S/N ratio > 10, sensitivity of 520%/ppm

**Table 3:** Ammonia gas sensing TMDC materials and their performances.

Material	Target Gas	LOD	Condition	Remarks
MoSe_2_ [149]	NH_3_	50 ppm	RT	Response time of 2.5 min, recovery time of 9 min.
Graphene/MoS_2_ [146]	NH_3_	5 ppm	150 °C	Response time < 10 min, Recovery time < 30 min.
WS_2_ [148]	NH_3_	50 ppm	RT	Response time of 200 s, recovery time of 232.3 s.
MoS_2_ [121]	NH_3_	1 ppm	RT	Response time 5–9 min, recovery time < 15 min.
UV-treated MoS_2_ [139]	NH_3_	100 ppm	RT	Response time of 7 min, Recovery time of 12 min.
MoTe_2_ [147]	NH_3_	2 ppm	RT	Over 95% recovery using gate biases of 0 V and 20 V. Response time 10 min, recovery time 20 min.
MoS_2_/Co_3_O_4_ [150]	NH_3_	0.1 ppm	RT	Response time is 98 s, recovery time is 100 s.
PMMA-MoS_2_ [151]	NH_3_	1 ppm	RT	Sensitivity of 54%, response time of 10 s, recovery time of 14 s.
MoS_2_/VS_2_ [152]	NH_3_	5 ppm	40 °C	Recovery and response time both < 5 min.

**Table 4:** Application of TMDCs in CO_2_ conversion to syngas and other gases.

Mechanism	Catalyst/Material	Details	Condition	Product
Electro-catalyst	2D nanoflake WSe_2_ [255]	50 vol% EMIM-BF4 in water	−0.764 V	CO
Electro-catalyst	Hierarchical MoS_x_Se_(2 − x)_ hybrid nanostructures [256]	0.1 M H_2_SO_4_ solution	−0.70V	H_2_
Electro-catalyst	^1^ Monolayer MoS_2_ [257]	-	-	CO
Electro-catalyst	5% niobium (Nb)-doped vertically aligned MoS_2_ [258]	50 vol% EMIM-BF_4_ in water	−0.8 V	CO
Electro-catalyst	molybdenum disulfide nanoflakes (MoS_2_ NFs) [259]	2.0 M C_5_H_14_ClNO	−2.0 V	CO
Electro-catalyst	Ultrathin MoTe_2_ [260]	0.1 M KHCO_3_	–1.0 V	CH_4_
Photo-catalyst	MoS_2_ nanoplatelet supported on few layer graphene [261]	Up to 60% conversion, 90% CH_4_ selectivity	React with H_2_, 250–500 °C	CH_4_, CO
Photo-catalyst	Marigold-like Si@MoS_2_[254]	Produced 323 μL∙g^−1^∙h^−1^ CH_4_, 23 μL∙g^−1^∙h^−1^ O_2_	React with H_2_O	CH_4_, O_2_
Photo-catalyst	Mesoporous TiO_2_ on 3D Graphene with Few-layered MoS_2_[262]	CO selectivity of 97% and yield of 93.22 µmol/g h	-	CO
Photo-catalyst	Z-scheme MoS_2_/g-C_3_N_4_ heterojunction [263]	Produced 58.59 μmol∙g^−1^ in 7 h.	25 °C, 100 kPa	CO
Photo-catalyst	MoS_2_/TiO_2_ heterojunction [264]	Produced 268.97 μmol∙g^−1^ CO and 49.93 μmol∙g^−1^ CH_4_	25 °C, 100 kPa	CO, CH_4_

^1^ Computational simulation using density functional theory (DFT).

**Table 5:** Application of TMDCs in converting CO_2_ to high-value chemicals.

Mechanism	Catalyst/Material	Details	Condition	Product
Photo-catalyst	Undecorated 2D-MoS_2_ [249]	Produced 27.4 μmol∙g^−1^∙h^−1^ methanol and 2.2 μmol∙g^−1^∙h^−1^ acetaldehyde	0.5 M NaHCO_3_	Methanol, acetaldehyde
Photo-catalyst	MoS_2_/Bi_2_WO_6_ nanocomposites [277]	Produced 36.7 μmol∙g^−1^ methanol and 36.6 μmol∙g^−1^ ethanol (in 4 h)	Deionized water	Methanol, ethanol
Photoelectro-catalyst	Co-doped MoS_2_ NPs [278]	Produced 35 mmol∙L^−1^ methanol (in 350 min)	−0.64 V	Methanol
Photo-catalyst	Nano Ag decorated MoS_2_ nanosheets [271]	Produced 365.08 μmol∙g^−1^∙h^−1^	20 mL isopropanol,	Methanol
Electro-catalysis	MoS_2_ electrodes [275]	0.1 M potassium phosphate buffer	−0.59 V	1-propanol
Photo-Catalyst	WSe_2_/Graphene/TiO_2_ nanocomposite [279]	Produced 6.326 μmol∙g^−1^∙h^−1^ methanol	Distilled water with Na_2_SO_3_ reagent	Methanol
Photo-catalyst	MoS_2_/TiO_2_[273]	Produced 10.6 μmol∙g^−1^∙h^−1^ methanol	Distilled Water and methanol	Methanol
Photo-catalyst	WS_2_ quantum dots doped Bi_2_S_3_ nanotubes [276]	Produced 38.2 μmol∙g^−1^∙h^−1^ methanol and 27.8 μmol∙g^−1^∙h^−1^ ethanol	Ultrapure water	Methanol, ethanol

## References

[1] Radisavljevic B., Radenovic A., Brivio J., Giacometti V., Kis A. (2011). Single-layer MoS_2_ transistors. Nat. Nanotechnol..

[2] Yin Z., Li H., Li H., Jiang L., Shi Y., Sun Y., Lu G., Zhang Q., Chen X., Zhang H. (2012). Single-layer MoS_2_ phototransistors. ACS Nano.

[3] Mak K.F., McGill K.L., Park J., McEuen P.L. (2014). The valley hall effect in MoS_2_ transistors. Science.

[4] Li H., Jia X., Zhang Q., Wang X. (2018). Metallic transition-metal dichalcogenide nanocatalysts for energy conversion. Chem.

[5] Gao Y.-P., Wu X., Huang K.-J., Xing L.-L., Zhang Y.-Y., Liu L. (2017). Two-dimensional transition metal diseleniums for energy storage application: A review of recent developments. CrystEngComm.

[6] Lu J.M., Zheliuk O., Leermakers I., Yuan N.F.Q., Zeitler U., Law K.T., Ye J.T. (2015). Evidence for two-dimensional Ising superconductivity in gated MoS_2_. Science.

[7] Liu X., Zhang Y.W. (2018). Thermal properties of transition-metal dichalcogenide. Chin. Phys. B.

[8] Kappera R., Voiry D., Yalcin S.E., Branch B., Gupta G., Mohite A.D., Chhowalla M. (2014). Phase-engineered low-resistance contacts for ultrathin MoS_2_ transistors. Nat. Mater..

[9] Kolobov A.V., Tominaga J. (2016). Two-Dimensional Transition-Metal Dichalcogenides.

[10] Wang Q.H., Kalantar-Zadeh K., Kis A., Coleman J.N., Strano M.S. (2012). Electronics and optoelectronics of two-dimensional transition metal dichalcogenides. Nat. Nanotechnol..

[11] Santosh K.C., Longo R.C., Addou R., Wallace R.M., Cho K. (2014). Impact of intrinsic atomic defects on the electronic structure of MoS_2_ monolayers. Nanotechnology.

[12] Feng S., Lin Z., Gan X., Lv R., Terrones M. (2017). Doping two-dimensional materials: Ultra-sensitive sensors, band gap tuning and ferromagnetic monolayers. Nanoscale Horizons.

[13] Ghorbani-Asl M., Borini S., Kuc A., Heine T. (2013). Strain-dependent modulation of conductivity in single-layer transition-metal dichalcogenides. Phys. Rev. B Condens. Matter Mater. Phys..

[14] Su X., Ju W., Zhang R., Guo C., Zheng J., Yong Y., Li X. (2016). Bandgap engineering of MoS_2_/MX_2_ (MX_2_ = WS_2_, MoSe_2_ and WSe_2_) heterobilayers subjected to biaxial strain and normal compressive strain. RSC Adv..

[15] Gusakova J., Wang X., Shiau L.L., Krivosheeva A., Shaposhnikov V., Borisenko V., Gusakov V., Tay B.K. (2017). Electronic properties of bulk and monolayer TMDs: Theoretical study within DFT framework (GVJ-2e method). Phys. Status Solidi Appl. Mater. Sci..

[16] Mak K.F., Lee C., Hone J., Shan J., Heinz T.F. (2010). Atomically thin MoS_2_: A new direct-gap semiconductor. Phys. Rev. Lett..

[17] Acerce M., Voiry D., Chhowalla M. (2015). Metallic 1T phase MoS_2_ nanosheets as supercapacitor electrode materials. Nat. Nanotechnol..

[18] Li H., Tan Y., Liu P., Guo C., Luo M., Han J., Lin T., Huang F., Chen M. (2016). Atomic-sized pores enhanced electrocatalysis of TaS_2_ nanosheets for hydrogen evolution. Adv. Mater..

[19] Stephenson T., Li Z., Olsen B., Mitlin D. (2014). Lithium ion battery applications of molybdenum disulfide (MoS_2_) nanocomposites. Energy Environ. Sci..

[20] Chang Y.H., Lin C.T., Chen T.Y., Hsu C.L., Lee Y.H., Zhang W., Wei K.H., Li L.J. (2013). Highly efficient electrocatalytic hydrogen production by MoS_x_ grown on graphene-protected 3D Ni foams. Adv. Mater..

[21] Kannan P.K., Late D.J., Morgan H., Rout C.S. (2015). Recent developments in 2D layered inorganic nanomaterials for sensing. Nanoscale.

[22] Sun Y., Gao S., Lei F., Xie Y. (2015). Atomically-thin two-dimensional sheets for understanding active sites in catalysis. Chem. Soc. Rev..

[23] Sang Y., Zhao Z., Zhao M., Hao P., Leng Y., Liu H. (2015). From UV to near-infrared, WS_2_ nanosheet: A novel photocatalyst for full solar light spectrum photodegradation. Adv. Mater..

[24] Atkin P., Daeneke T., Wang Y., Carey B.J., Berean K.J., Clark R.M., Ou J.Z., Trinchi A., Cole I.S., Kalantar-Zadeh K. (2016). 2D WS_2_/carbon dot hybrids with enhanced photocatalytic activity. J. Mater. Chem. A.

[25] Di J., Xia J., Ge Y., Xu L., Xu H., Chen J., He M., Li H. (2014). Facile fabrication and enhanced visible light photocatalytic activity of few-layer MoS_2_ coupled BiOBr microspheres. Dalt. Trans..

[26] Jaramillo T.F., Jørgensen K.P., Bonde J., Nielsen J.H., Horch S., Chorkendorff I. (2007). Identification of active edge sites for electrochemical H_2_ evolution from MoS_2_ nanocatalysts. Science.

[27] Mohanty B., Ghorbani-Asl M., Kretschmer S., Ghosh A., Guha P., Panda S.K., Jena B., Krasheninnikov A.V., Jena B.K. (2018). MoS_2_ quantum dots as efficient catalyst materials for the oxygen evolution reaction. ACS Catal..

[28] Ye G., Gong Y., Lin J., Li B., He Y., Pantelides S.T., Zhou W., Vajtai R., Ajayan P.M. (2016). Defects engineered monolayer MoS_2_ for improved hydrogen evolution reaction. Nano Lett..

[29] Chan K., Tsai C., Hansen H.A., Nørskov J.K. (2014). Molybdenum sulfides and selenides as possible electrocatalysts for CO_2_ reduction. ChemCatChem.

[30] Mohajerin T.J., Helz G.R., Johannesson K.H. (2016). Tungsten-molybdenum fractionation in estuarine environments. Geochim. Cosmochim. Acta.

[31] Ren Z., Zhou T., Hollings P., White N.C., Wang F., Yuan F. (2018). Trace element geochemistry of molybdenite from the Shapinggou super-large porphyry Mo deposit, China. Ore Geol. Rev..

[32] Golden J., McMillan M., Downs R.T., Hystad G., Goldstein I., Stein H.J., Zimmerman A., Sverjensky D.A., Armstrong J.T., Hazen R.M. (2013). Rhenium variations in molybdenite (MoS_2_): Evidence for progressive subsurface oxidation. Earth Planet. Sci. Lett..

[33] Pumera M., Sofer Z., Ambrosi A. (2014). Layered transition metal dichalcogenides for electrochemical energy generation and storage. J. Mater. Chem. A.

[34] Eftekhari A. (2017). Tungsten dichalcogenides (WS_2_, WSe_2_, and WTe_2_): Materials chemistry and applications. J. Mater. Chem. A.

[35] Zhan Y., Liu Z., Najmaei S., Ajayan P.M., Lou J. (2012). Large-area vapor-phase growth and characterization of MoS_2_ atomic layers on a SiO_2_ substrate. Small.

[36] Ji Q., Li C., Wang J., Niu J., Gong Y., Zhang Z., Fang Q., Zhang Y., Shi J., Liao L. (2017). Metallic vanadium disulfide nanosheets as a platform material for multifunctional electrode applications. Nano Lett..

[37] Wilson J.A., Yoffe A.D. (1969). The transition metal dichalcogenides discussion and interpretation of the observed optical, electrical and structural properties. Adv. Phys..

[38] Ubaldini A., Jacimovic J., Ubrig N., Giannini E. (2013). Chloride-driven chemical vapor transport method for crystal growth of transition metal dichalcogenides. Cryst. Growth Des..

[39] Zhang X., Lou F., Li C., Zhang X., Jia N., Yu T., He J., Zhang B., Xia H., Wang S. (2015). Flux method growth of bulk MoS_2_ single crystals and their application as a saturable absorber. CrystEngComm.

[40] Ye L., Xu H., Zhang D., Chen S. (2014). Synthesis of bilayer MoS_2_ nanosheets by a facile hydrothermal method and their methyl orange adsorption capacity. Mater. Res. Bull..

[41] Kalosi A., Demydenko M., Bodik M., Hagara J., Kotlar M., Kostiuk D., Halahovets Y., Vegso K., Marin Roldan A., Maurya G.S. (2019). Tailored langmuir–schaefer deposition of few-layer MoS_2_ nanosheet films for electronic applications. Langmuir.

[42] Huang X., Zeng Z., Zhang H. (2013). Metal dichalcogenide nanosheets: Preparation, properties and applications. Chem. Soc. Rev..

[43] Li H., Wu J., Yin Z., Zhang H. (2014). Preparation and applications of mechanically exfoliated single-layer and multilayer MoS_2_ and WSe_2_ nanosheets. Acc. Chem. Res..

[44] Cunningham G., Lotya M., Cucinotta C.S., Sanvito S., Bergin S.D., Menzel R., Shaffer M.S.P., Coleman J.N. (2012). Solvent exfoliation of transition metal dichalcogenides: Dispersibility of exfoliated nanosheets varies only weakly between compounds. ACS Nano.

[45] Bissett M.A., Worrall S.D., Kinloch I.A., Dryfe R.A.W. (2016). Comparison of two-dimensional transition metal dichalcogenides for electrochemical supercapacitors. Electrochim. Acta.

[46] Yue Q., Shao Z., Chang S., Li J. (2013). Adsorption of gas molecules on monolayer MoS_2_ and effect of applied electric field. Nanoscale Res. Lett..

[47] Kumar R., Goel N., Hojamberdiev M., Kumar M. (2020). Transition metal dichalcogenides-based flexible gas sensors. Sensors Actuators A Phys..

[48] Elsevier Welcome to Scopus Preview. https://www.scopus.com/home.uri.

[49] Blumenthal I. (2001). Carbon monoxide poisoning. J. R. Soc. Med..

[50] Li Z., He J., Wang H., Wang B., Ma X. (2015). Enhanced methanation stability of nano-sized MoS_2_ catalysts by adding Al_2_O_3_. Front. Chem. Sci. Eng..

[51] Almeida K., Peña P., Rawal T.B., Coley W.C., Akhavi A.A., Wurch M., Yamaguchi K., Le D., Rahman T.S., Bartels L. (2019). A single layer of MoS_2_ activates gold for room temperature CO oxidation on an inert silica substrate. J. Phys. Chem. C.

[52] Lolla S., Luo X. (2020). Tuning the catalytic properties of monolayer MoS_2_ through doping and sulfur vacancies. Appl. Surf. Sci..

[53] Ma D., Tang Y., Yang G., Zeng J., He C., Lu Z. (2015). CO catalytic oxidation on iron-embedded monolayer MoS_2_. Appl. Surf. Sci..

[54] Lu X., Guo L., Wang P., Cui M., Kanghong D., Peng W. (2020). Theoretical investigation of the adsorption of gas molecules on WSe_2_ monolayers decorated with Pt, Au nanoclusters. Appl. Surf. Sci..

[55] Cui H., Zhang G., Zhang X., Tang J. (2019). Rh-doped MoSe_2_ as a toxic gas scavenger: A first-principles study. Nanoscale Adv..

[56] Chen W., Zijlstra B., Filot I.A.W., Pestman R., Hensen E.J.M. (2018). Mechanism of carbon monoxide dissociation on a cobalt Fischer–Tropsch catalyst. ChemCatChem.

[57] American Chemical Society (ACS) It’s Water Vapor, Not the CO2. https://www.acs.org/content/acs/en/climatescience/climatesciencenarratives/its-water-vapor-not-the-co2.html.

[58] Kong D., Wang H., Cha J.J., Pasta M., Koski K.J., Yao J., Cui Y. (2013). Synthesis of MoS_2_ and MoSe_2_ films with vertically aligned layers. Nano Lett..

[59] Wang H., Kong D., Johanes P., Cha J.J., Zheng G., Yan K., Liu N., Cui Y. (2013). MoSe_2_ and WSe_2_ nanofilms with vertically aligned molecular layers on curved and rough surfaces. Nano Lett..

[60] Hinnemann B., Moses P.G., Bonde J., Jørgensen K.P., Nielsen J.H., Horch S., Chorkendorff I., Nørskov J.K. (2005). Biomimetic hydrogen evolution: MoS_2_ nanoparticles as catalyst for hydrogen evolution. J. Am. Chem. Soc..

[61] Yu K., Groom D.J., Wang X., Yang Z., Gummalla M., Ball S.C., Myers D.J., Ferreira P.J. (2014). Degradation mechanisms of platinum nanoparticle catalysts in proton exchange membrane fuel cells: The role of particle size. Chem. Mater..

[62] Zhao Y., Hwang J., Tang M.T., Chun H., Wang X., Zhao H., Chan K., Han B., Gao P., Li H. (2020). Ultrastable molybdenum disulfide-based electrocatalyst for hydrogen evolution in acidic media. J. Power Sources.

[63] Rong H., Zhang H., Xiao S., Li C., Hu C. (2016). Optimizing energy consumption for data centers. Renew. Sustain. Energy Rev..

[64] Li B.B., Qiao S.Z., Zheng X.R., Yang X.J., Cui Z.D., Zhu S.L., Li Z.Y., Liang Y.Q. (2015). Pd coated MoS_2_ nanoflowers for highly efficient hydrogen evolution reaction under irradiation. J. Power Sources.

[65] Jian J., Li Y., Bi H., Wang X., Wu X., Qin W. (2020). Aluminum decoration on MoS_2_ ultrathin nanosheets for highly efficient hydrogen evolution. ACS Sustain. Chem. Eng..

[66] Bar-Ziv R., Ranjan P., Lavie A., Jain A., Garai S., Bar Hen A., Popovitz-Biro R., Tenne R., Arenal R., Ramasubramaniam A. (2019). Au-MoS_2_ hybrids as hydrogen evolution electrocatalysts. ACS Appl. Energy Mater..

[67] Chia X., Sutrisnoh N.A.A., Pumera M. (2018). Tunable Pt-MoS_x_ hybrid catalysts for hydrogen evolution. ACS Appl. Mater. Interfaces.

[68] Rastogi P.K., Sarkar S., Mandler D. (2017). Ionic strength induced electrodeposition of two-dimensional layered MoS_2_ nanosheets. Appl. Mater. Today.

[69] Cao J., Zhou J., Zhang Y., Liu X. (2017). A Clean and facile synthesis strategy of MoS_2_ nanosheets grown on multi-wall CNTs for enhanced hydrogen evolution reaction performance. Sci. Rep..

[70] Chua X.J., Pumera M. (2017). The effect of varying solvents for MoS_2_ treatment on its catalytic efficiencies for HER and ORR. Phys. Chem. Chem. Phys..

[71] Ren J., Zong H., Sun Y., Gong S., Feng Y., Wang Z., Hu L., Yu K., Yu K. (2020). 2D organ-like molybdenum carbide (MXene) coupled with MoS_2_ nanoflowers enhances the catalytic activity in the hydrogen evolution reaction. CrystEngComm.

[72] Wang Y., Jian C., He X., Liu W. (2020). Self-supported molybdenum selenide nanosheets grown on urchin-like cobalt selenide nanowires array for efficient hydrogen evolution. Int. J. Hydrogen Energy.

[73] Seok J., Lee J.H., Cho S., Ji B., Kim H.W., Kwon M., Kim D., Kim Y.M., Oh S.H., Kim S.W. (2017). Active hydrogen evolution through lattice distortion in metallic MoTe_2_. 2D Mater..

[74] Pan Y., Zheng F., Wang X., Qin H., Liu E., Sha J., Zhao N., Zhang P., Ma L. (2020). Enhanced electrochemical hydrogen evolution performance of WS_2_ nanosheets by Te doping. J. Catal..

[75] Kadam S.R., Enyashin A.N., Houben L., Bar-Ziv R., Bar-Sadan M. (2020). Ni-WSe_2_ nanostructures as efficient catalysts for electrochemical hydrogen evolution reaction (HER) in acidic and alkaline media. J. Mater. Chem. A.

[76] Rahman F.A., Aziz M.M.A., Saidur R., Bakar W.A.W.A., Hainin M., Putrajaya R., Hassan N.A. (2017). Pollution to solution: Capture and sequestration of carbon dioxide (CO_2_) and its utilization as a renewable energy source for a sustainable future. Renew. Sustain. Energy Rev..

[77] Shen Y., Wang H., Zhang X., Zhang Y. (2016). MoS_2_ nanosheets functionalized composite mixed matrix membrane for enhanced CO_2_ capture via surface drop-coating method. ACS Appl. Mater. Interfaces.

[78] Sun Q., Qin G., Ma Y., Wang W., Li P., Du A., Li Z. (2017). Electric field controlled CO_2_ capture and CO_2_/N_2_ separation on MoS_2_ monolayers. Nanoscale.

[79] Aguilar N., Aparicio S. (2019). Theoretical insights into CO_2_ adsorption by MoS_2_ nanomaterials. J. Phys. Chem. C.

[80] Shi G., Yu L., Ba X., Zhang X., Zhou J., Yu Y. (2017). Copper nanoparticle interspersed MoS_2_ nanoflowers with enhanced efficiency for CO_2_ electrochemical reduction to fuel. Dalt. Trans..

[81] Kim R., Kim J., Do J.Y., Seo M.W., Kang M. (2019). Carbon dioxide photoreduction on the Bi_2_S_3_/MoS_2_ catalyst. Catalysts.

[82] Hu X., Yang H., Gao M., Tian H., Li Y., Liang Z., Jian X. (2019). Insights into the photoassisted electrocatalytic reduction of CO_2_ over a two-dimensional MoS_2_ nanostructure loaded on SnO_2_ nanoparticles. ChemElectroChem.

[83] Yu L., Xie Y., Zhou J., Li Y., Yu Y., Ren Z. (2018). Robust and selective electrochemical reduction of CO_2_: The case of integrated 3D TiO_2_@MoS_2_ architectures and Ti-S bonding effects. J. Mater. Chem. A.

[84] Ai W., Kou L., Hu X., Wang Y., Krasheninnikov A.V., Sun L., Shen X. (2019). Enhanced sensitivity of MoSe_2_ monolayer for gas adsorption induced by electric field. J. Phys. Condens. Matter.

[85] Rathi K., Pal K. (2020). Ruthenium-decorated tungsten disulfide quantum dots for a CO_2_ gas sensor. Nanotechnology.

[86] Myllyvirta L. Global Air Pollution Map: Ranking the World’s Worst SO2 and NO2 Emission Hotspots. https://storage.googleapis.com/planet4-africa-stateless/2019/03/625c2655-ranking-so2-and-no2-hotspots_19-march-2019.pdf.

[87] Aas W., Mortier A., Bowersox V., Cherian R., Faluvegi G., Fagerli H., Hand J., Klimont Z., Galy-Lacaux C., Lehmann C.M.B. (2019). Global and regional trends of atmospheric sulfur. Sci. Rep..

[88] Ye X., Ma S., Jiang X., Yang Z., Jiang W., Wang H. (2019). The adsorption of acidic gaseous pollutants on metal and nonmetallic surface studied by first-principles calculation: A review. Chinese Chem. Lett..

[89] Gui Y., Chen J., Wang W., Zhu Y., Tang C., Xu L. (2019). Adsorption mechanism of hydrogen sulfide and sulfur dioxide on Au–MoS_2_ monolayer. Superlattices Microstruct..

[90] Qian H., Lu W., Wei X., Chen W., Deng J. (2019). H_2_S and SO_2_ adsorption on Pt-MoS_2_ adsorbent for partial discharge elimination: A DFT study. Results Phys..

[91] Yang Y., Ashraf M.A., Jermsittiparsert K., Jiang L., Zhang D. (2020). Enhancing the adsorption performance and sensing capability of Ti-doped MoSe_2_ and MoS_2_ monolayers by applying electric field. Appl. Surf. Sci..

[92] Ma S., Su L., Jin L., Su J., Jin Y. (2019). A first-principles insight into Pd-doped MoSe_2_ monolayer: A toxic gas scavenger. Phys. Lett. A.

[93] Abbasi A., Sardroodi J.J. (2017). A novel strategy for SO_x_ removal by N-doped TiO_2_/WSe_2_ nanocomposite as a highly efficient molecule sensor investigated by van der Waals corrected DFT. Comput. Theor. Chem..

[94] Ni J., Wang W., Quintana M., Jia F., Song S. (2020). Adsorption of small gas molecules on strained monolayer WSe_2_ doped with Pd, Ag, Au, and Pt: A computational investigation. Appl. Surf. Sci..

[95] Dan M., Xiang J., Wu F., Yu S., Cai Q., Ye L., Ye Y., Zhou Y. (2019). Rich active-edge-site MoS_2_ anchored on reduction sites in metal sulfide heterostructure: Toward robust visible light photocatalytic hydrogen sulphide splitting. Appl. Catal. B Environ..

[96] Li Y., Yu S., Doronkin D.E., Wei S., Dan M., Wu F., Ye L., Grunwaldt J.D., Zhou Y. (2019). Highly dispersed PdS preferably anchored on In_2_S_3_ of MnS/In_2_S_3_ composite for effective and stable hydrogen production from H_2_S. J. Catal..

[97] Yuan Y., Wang W., Shi Y., Song L., Ma C., Hu Y. (2020). The influence of highly dispersed Cu_2_O-anchored MoS_2_ hybrids on reducing smoke toxicity and fire hazards for rigid polyurethane foam. J. Hazard. Mater..

[98] Wen M.Q., Xiong T., Zang Z.G., Wei W., Tang X.S., Dong F. (2016). Synthesis of MoS_2_/g-C_3_N_4_ nanocomposites with enhanced visible-light photocatalytic activity for the removal of nitric oxide (NO). Opt. Express.

[99] Xiong T., Wen M., Dong F., Yu J., Han L., Lei B., Zhang Y., Tang X., Zang Z. (2016). Three dimensional Z-scheme (BiO)_2_CO_3_/MoS_2_ with enhanced visible light photocatalytic NO removal. Appl. Catal. B Environ..

[100] Hu J., Chen D., Li N., Xu Q., Li H., He J., Lu J. (2017). In situ fabrication of Bi_2_O_2_CO_3_/MoS_2_ on carbon nanofibers for efficient photocatalytic removal of NO under visible-light irradiation. Appl. Catal. B Environ..

[101] Zhu J., Zhang H., Tong Y., Zhao L., Zhang Y., Qiu Y., Lin X. (2017). First-principles investigations of metal (V, Nb, Ta)-doped monolayer MoS_2_: Structural stability, electronic properties and adsorption of gas molecules. Appl. Surf. Sci..

[102] Ovcharenko R., Dedkov Y., Voloshina E. (2016). Adsorption of NO_2_ on WSe_2_: DFT and photoelectron spectroscopy studies. J. Phys. Condens. Matter.

[103] Ala-Mantila S., Heinonen J., Junnila S. (2014). Relationship between urbanization, direct and indirect greenhouse gas emissions, and expenditures: A multivariate analysis. Ecol. Econ..

[104] Denmead O.T., Chen D., Griffith D.W.T., Loh Z.M., Bai M., Naylor T. (2008). Emissions of the indirect greenhouse gases NH_3_ and NO_x_ from Australian beef cattle feedlots. Aust. J. Exp. Agric..

[105] Tai A.P.K., Martin M.V., Heald C.L. (2014). Threat to future global food security from climate change and ozone air pollution. Nat. Clim. Chang..

[106] Tjoelker M.G., Volin J.C., Oleksyn J., Reich P.B. (1995). Interaction of ozone pollution and light effects on photosynthesis in a forest canopy experiment. Plant. Cell Environ..

[107] Hulin M., Simoni M., Viegi G., Annesi-Maesano I. (2012). Respiratory health and indoor air pollutants based on quantitative exposure assessments. Eur. Respir. J..

[108] Ajugwo A.O. (2013). Negative effects of gas flaring: The Nigerian experience. J. Environ. Pollut. Hum. Health.

[109] Collins F., Orpen D., Fay C., Foley C., Smeaton A.F., Diamond D. Web-based monitoring of year-length deployments of autonomous gas sensing platforms on landfill sites. Proceedings of the 2011 IEEE Sensors.

[110] Rossi M., Brunelli D., Adami A., Lorenzelli L., Menna F., Remondino F. Gas-drone: Portable gas sensing system on UAVs for gas leakage localization. Proceedings of the 2014 IEEE Sensors.

[111] Li L., Fu C., Lou Z., Chen S., Han W., Jiang K., Chen D., Shen G. (2017). Flexible planar concentric circular micro-supercapacitor arrays for wearable gas sensing application. Nano Energy.

[112] Suh J.H., Cho I., Kang K., Kweon S.J., Lee M., Yoo H.J., Park I. (2018). Fully integrated and portable semiconductor-type multi-gas sensing module for IoT applications. Sensors Actuators B Chem..

[113] Cho B., Hahm M.G., Choi M., Yoon J., Kim A.R., Lee Y.J., Park S.G., Kwon J.D., Kim C.S., Song M. (2015). Charge-transfer-based gas sensing using atomic-layer MoS_2_. Sci. Rep..

[114] Liu L., Ikram M., Ma L., Zhang X., Lv H., Ullah M., Khan M., Yu H., Shi K. (2020). Edge-exposed MoS_2_ nanospheres assembled with SnS_2_ nanosheet to boost NO_2_ gas sensing at room temperature. J. Hazard. Mater..

[115] Pham T., Li G., Bekyarova E., Itkis M.E., Mulchandani A. (2019). MoS_2_-based optoelectronic gas sensor with sub-parts-per-billion limit of NO_2_ gas detection. ACS Nano.

[116] Guo S., Yang D., Zhang S., Dong Q., Li B., Tran N., Li Z., Xiong Y., Zaghloul M.E. (2019). Development of a cloud-based epidermal MoSe_2_ device for hazardous gas sensing. Adv. Funct. Mater..

[117] Zhang Q., Shen Z., Cao J., Zhang R., Zhang L., Huang R.J., Zheng C., Wang L., Liu S., Xu H. (2015). Variations in PM_2.5_, TSP, BC, and trace gases (NO_2_, SO_2_, and O_3_) between haze and non-haze episodes in winter over Xi’an, China. Atmos. Environ..

[118] Pijolat C., Pupier C., Sauvan M., Tournier G., Lalauze R. (1999). Gas detection for automotive pollution control. Sensors Actuators B Chem..

[119] Yamazoe N., Miura N. (1994). Environmental gas sensing. Sensors Actuators B Chem..

[120] Long H., Harley-Trochimczyk A., Pham T., Tang Z., Shi T., Zettl A., Carraro C., Worsley M.A., Maboudian R. (2016). High surface area MoS_2_/graphene hybrid aerogel for ultrasensitive NO_2_ detection. Adv. Funct. Mater..

[121] Liu B., Chen L., Liu G., Abbas A.N., Fathi M., Zhou C. (2014). High-performance chemical sensing using Schottky-contacted chemical vapor deposition grown monolayer MoS_2_ transistors. ACS Nano.

[122] Yan W., Worsley M.A., Pham T., Zettl A., Carraro C., Maboudian R. (2018). Effects of ambient humidity and temperature on the NO_2_ sensing characteristics of WS_2_/graphene aerogel. Appl. Surf. Sci..

[123] Zhou Y., Liu G., Zhu X., Guo Y. (2017). Ultrasensitive NO_2_ gas sensing based on rGO/MoS_2_ nanocomposite film at low temperature. Sensors Actuators B Chem..

[124] Mirõ P., Ghorbani-Asl M., Heine T. (2014). Two dimensional materials beyond MoS_2_: Noble-transition-metal dichalcogenides. Angew. Chemie Int. Ed..

[125] Sajjad M., Montes E., Singh N., Schwingenschlögl U. (2017). Superior gas sensing properties of monolayer PtSe_2_. Adv. Mater. Interfaces.

[126] Yim C., Lee K., McEvoy N., O’Brien M., Riazimehr S., Berner N.C., Cullen C.P., Kotakoski J., Meyer J.C., Lemme M.C. (2016). High-performance hybrid electronic devices from layered PtSe_2_ films grown at low temperature. ACS Nano.

[127] Wu E., Xie Y., Yuan B., Zhang H., Hu X., Liu J., Zhang D. (2018). Ultrasensitive and fully reversible NO_2_ gas sensing based on p-type MoTe_2_ under ultraviolet illumination. ACS Sensors.

[128] Ko K.Y., Song J.G., Kim Y., Choi T., Shin S., Lee C.W., Lee K., Koo J., Lee H., Kim J. (2016). Improvement of gas-sensing performance of large-area tungsten disulfide nanosheets by surface functionalization. ACS Nano.

[129] Cho B., Kim A.R., Park Y., Yoon J., Lee Y.J., Lee S., Yoo T.J., Kang C.G., Lee B.H., Ko H.C. (2015). Bifunctional sensing characteristics of chemical vapor deposition synthesized atomic-layered MoS_2_. ACS Appl. Mater. Interfaces.

[130] Wang J., Deng J., Li Y., Yuan H., Xu M. (2020). ZnO nanocrystal-coated MoS_2_ nanosheets with enhanced ultraviolet light gas sensitive activity studied by surface photovoltage technique. Ceram. Int..

[131] Baek J., Yin D., Liu N., Omkaram I., Jung C., Im H., Hong S., Kim S.M., Hong Y.K., Hur J. (2017). A highly sensitive chemical gas detecting transistor based on highly crystalline CVD-grown MoSe_2_ films. Nano Res..

[132] Perrozzi F., Emamjomeh S.M., Paolucci V., Taglieri G., Ottaviano L., Cantalini C. (2017). Thermal stability of WS_2_ flakes and gas sensing properties of WS_2_/WO_3_ composite to H_2_, NH_3_ and NO_2_. Sensors Actuators B Chem..

[133] Han S., Bian H., Feng Y., Liu A., Li X., Zeng F., Zhang X. (2011). Analysis of the relationship between O_3_, NO and NO_2_ in Tianjin, China. Aerosol Air Qual. Res..

[134] Jones A.E., Weller R., Wolff E.W., Jacobi H.W. (2000). Speciation and rate of photochemical NO and NO_2_ production in Antarctic snow. Geophys. Res. Lett..

[135] Environmental Protection Agency (EPA) (1999). Nitrogen Oxides (NOx), Why and How They Are Controlled.

[136] Zhao S., Xue J., Kang W. (2014). Gas adsorption on MoS_2_ monolayer from first-principles calculations. Chem. Phys. Lett..

[137] Li H., Yin Z., He Q., Li H., Huang X., Lu G., Fam D.W.H., Tok A.I.Y., Zhang Q., Zhang H. (2012). Fabrication of single- and multilayer MoS_2_ film-based field-effect transistors for sensing NO at room temperature. Small.

[138] Ramu S., Chandrakalavathi T., Murali G., Kumar K.S., Sudharani A., Ramanadha M., Peta K.R., Jeyalakshmi R., Vijayalakshmi R.P. (2019). UV enhanced NO gas sensing properties of the MoS_2_ monolayer gas sensor. Mater. Res. Express.

[139] Burman D., Ghosh R., Santra S., Kumar Ray S., Kumar Guha P. (2017). Role of vacancy sites and UV-ozone treatment on few layered MoS_2_ nanoflakes for toxic gas detection. Nanotechnology.

[140] Medina H., Li J.G., Su T.Y., Lan Y.W., Lee S.H., Chen C.W., Chen Y.Z., Manikandan A., Tsai S.H., Navabi A. (2017). Wafer-scale growth of WSe_2_ monolayers toward phase-engineered hybrid WO_x_/WSe_2_ films with sub-ppb NO_x_ gas sensing by a low-temperature plasma-assisted selenization process. Chem. Mater..

[141] Committee on the Environment and Natural Resources Air Quality Research Subcommittee (2000). Atmospheric Ammonia: Sources and Fate. A review of Ongoing Federal Research and Future Needs.

[142] Pinder R.W., Gilliland A.B., Dennis R.L. (2008). Environmental impact of atmospheric NH3 emissions under present and future conditions in the eastern United States. Geophys. Res. Lett..

[143] Donham K.J., Cumro D., Reynolds S. (2002). Synergistic effects of dust and ammonia on the occupational health effects of poultry production workers. J. Agromedicine.

[144] Guo S., Yang D., Li B., Dong Q., Li Z., Zaghloul M.E. An artificial intelligent flexible gas sensor based on ultra-large area MoSe_2_ nanosheet. Proceedings of the 2019 IEEE 62nd International Midwest Symposium on Circuits and Systems (MWSCAS).

[145] Luo H., Cao Y., Zhou J., Feng J., Cao J., Guo H. (2016). Adsorption of NO_2_, NH_3_ on monolayer MoS_2_ doped with Al, Si, and P: A first-principles study. Chem. Phys. Lett..

[146] Cho B., Yoon J., Lim S.K., Kim A.R., Kim D.H., Park S.G., Kwon J.D., Lee Y.J., Lee K.H., Lee B.H. (2015). Chemical sensing of 2D graphene/MoS_2_ heterostructure device. ACS Appl. Mater. Interfaces.

[147] Feng Z., Xie Y., Chen J., Yu Y., Zheng S., Zhang R., Li Q., Chen X., Sun C., Zhang H. (2017). Highly sensitive MoTe_2_ chemical sensor with fast recovery rate through gate biasing. 2D Mater..

[148] Qin Z., Zeng D., Zhang J., Wu C., Wen Y., Shan B., Xie C. (2017). Effect of layer number on recovery rate of WS_2_ nanosheets for ammonia detection at room temperature. Appl. Surf. Sci..

[149] Late D.J., Doneux T., Bougouma M. (2014). Single-layer MoSe_2_ based NH_3_ gas sensor. Appl. Phys. Lett..

[150] Zhang D., Jiang C., Li P., Sun Y. (2017). Layer-by-layer self-assembly of Co_3_O_4_ nanorod-decorated MoS_2_ nanosheet-based nanocomposite toward high-performance ammonia detection. ACS Appl. Mater. Interfaces.

[151] Abun A., Huang B.R., Saravanan A., Kathiravan D., Hong P. (2020). Da Effect of PMMA on the surface of exfoliated MoS_2_ nanosheets and their highly enhanced ammonia gas sensing properties at room temperature. J. Alloys Compd..

[152] Zhang S., Wang J., Torad N.L., Xia W., Aslam M.A., Kaneti Y.V., Hou Z., Ding Z., Da B., Fatehmulla A. (2020). Rational design of nanoporous MoS_2_/VS_2_ heteroarchitecture for ultrahigh performance ammonia sensors. Small.

[153] Durmusoglu E., Taspinar F., Karademir A. (2010). Health risk assessment of BTEX emissions in the landfill environment. J. Hazard. Mater..

[154] Dai H., Jing S., Wang H., Ma Y., Li L., Song W., Kan H. (2017). VOC characteristics and inhalation health risks in newly renovated residences in Shanghai, China. Sci. Total Environ..

[155] Cetin E., Odabasi M., Seyfioglu R. (2003). Ambient volatile organic compound (VOC) concentrations around a petrochemical complex and a petroleum refinery. Sci. Total Environ..

[156] Malherbe L., Mandin C. (2007). VOC emissions during outdoor ship painting and health-risk assessment. Atmos. Environ..

[157] Chen C., Tsow F., Campbell K.D., Iglesias R., Forzani E., Tao N. (2013). A wireless hybrid chemical sensor for detection of environmental volatile organic compounds. IEEE Sens. J..

[158] Sun X., Shao K., Wang T. (2016). Detection of volatile organic compounds (VOCs) from exhaled breath as noninvasive methods for cancer diagnosis. Anal. Bioanal. Chem..

[159] Barzegar M., Berahman M., Asgari R. (2019). First-principles study of molecule adsorption on Ni-decorated monolayer MoS2. J. Comput. Electron..

[160] Kim J.S., Yoo H.W., Choi H.O., Jung H.T. (2014). Tunable volatile organic compounds sensor by using thiolated ligand conjugation on MoS_2_. Nano Lett..

[161] Tomer V.K., Malik R., Chaudhary V., Mishra Y.K., Kienle L., Ahuja R., Lin L. (2019). Superior visible light photocatalysis and low-operating temperature VOCs sensor using cubic Ag(0)-MoS_2_ loaded g-CN 3D porous hybrid. Appl. Mater. Today.

[162] Zhao C., Gan X., Yuan Q., Hu S., Fang L., Zhao J. (2018). High-performance volatile organic compounds microsensor based on few-layer MoS_2_-coated photonic crystal cavity. Adv. Opt. Mater..

[163] Chen D., Tang J., Zhang X., Li Y., Liu H. (2019). Detecting decompositions of sulfur hexafluoride using MoS_2_ monolayer as gas sensor. IEEE Sens. J..

[164] Chen D., Zhang X., Tang J., Cui Z., Cui H., Pi S. (2018). Theoretical study of monolayer PtSe_2_ as outstanding gas sensor to detect SF_6_ decompositions. IEEE Electron Device Lett..

[165] Park J., Mun J., Shin J.S., Kang S.W. (2018). Highly sensitive two dimensional MoS_2_ gas sensor decorated with Pt nanoparticles. R. Soc. Open Sci..

[166] Yang Y., Jermsittiparsert K., Gao W., Zhang D. (2020). Structural, electronic and magnetic properties of the Ni/Cu-embedded S-vacancy defective MoS_2_ monolayers and their effects on the adsorption of SO_x_ and O_3_ molecules. Appl. Surf. Sci..

[167] Ma D., Ju W., Li T., Zhang X., He C., Ma B., Lu Z., Yang Z. (2016). The adsorption of CO and NO on the MoS_2_ monolayer doped with Au, Pt, Pd, or Ni: A first-principles study. Appl. Surf. Sci..

[168] Shen J., Yang Z., Wang Y., Xu L.C., Liu R., Liu X. (2020). The gas sensing performance of borophene/MoS_2_ heterostructure. Appl. Surf. Sci..

[169] Sahoo M.P.K., Wang J., Zhang Y., Shimada T., Kitamura T. (2016). Modulation of gas adsorption and magnetic properties of monolayer-MoS_2_ by antisite defect and strain. J. Phys. Chem. C.

[170] Ma D., Ma B., Lu Z., He C., Tang Y., Lu Z., Yang Z. (2017). Interaction between H_2_O, N_2_, CO, NO, NO_2_ and N_2_O molecules and a defective WSe_2_ monolayer. Phys. Chem. Chem. Phys..

[171] Jasmine J.M., Aadhityan A., Preferencial kala C., Thiruvadigal D.J. (2019). A first-principles study of Cl_2_, PH_3_, AsH_3_, BBr_3_ and SF_4_ gas adsorption on MoS_2_ monolayer with S and Mo vacancy. Appl. Surf. Sci..

[172] UNEP Tackling Global Water Pollution. https://www.unenvironment.org/explore-topics/water/what-we-do/tackling-global-water-pollution.

[173] Malik S.N., Khan S.M., Ghosh P.C., Vaidya A.N., Kanade G., Mudliar S.N. (2019). Treatment of pharmaceutical industrial wastewater by nano-catalyzed ozonation in a semi-batch reactor for improved biodegradability. Sci. Total Environ..

[174] Khan N.A., Khan K.A., Islam M. (2012). Water and wastewater treatment using nano-technology. Chemistry of Phytopotentials: Health, Energy and Environmental Perspectives.

[175] Prince R.C. (2015). Oil spill dispersants: Boon or bane?. Environ. Sci. Technol..

[176] Abiodun O.I., Jantan A., Omolara A.E., Dada K.V., Mohamed N.A., Arshad H. (2018). State-of-the-art in artificial neural network applications: A survey. Heliyon.

[177] Gusain R., Kumar N., Fosso-Kankeu E., Ray S.S. (2019). Efficient removal of Pb(II) and Cd(II) from industrial mine water by a hierarchical MoS_2_/SH-MWCNT nanocomposite. ACS Omega.

[178] Göktaş R.K., MacLeod M. (2016). Remoteness from sources of persistent organic pollutants in the multi-media global environment. Environ. Pollut..

[179] Wang Z., Wu A., Ciacchi L.C., Wei G. (2018). Recent advances in nanoporous membranes for water purification. Nanomaterials.

[180] SDG Knowledge Platform Sustainable Development Goals. https://sustainabledevelopment.un.org/?menu=1300.

[181] Lu H., Wang J., Stoller M., Wang T., Bao Y., Hao H. (2016). An overview of nanomaterials for water and wastewater treatment. Adv. Mater. Sci. Eng..

[182] Li G., Wang Y., Bi J., Huang X., Mao Y., Luo L., Hao H. (2020). Partial oxidation strategy to synthesize WS_2_/WO_3_ heterostructure with enhanced adsorption performance for organic dyes: Synthesis, modelling, and mechanism. Nanomaterials.

[183] Wang Z., Mi B. (2017). Environmental applications of 2D molybdenum disulfide (MoS_2_) nanosheets. Environ. Sci. Technol..

[184] Tang H., Huang H., Wang X., Wu K., Tang G., Li C. (2016). Hydrothermal synthesis of 3D hierarchical flower-like MoSe_2_ microspheres and their adsorption performances for methyl orange. Appl. Surf. Sci..

[185] Fang Y., Huang Q., Liu P., Shi J., Xu G. (2018). Easy-separative MoS_2_-glue sponges with high-efficient dye adsorption and excellent reusability for convenient water treatment. Colloids Surfaces A Physicochem. Eng. Asp..

[186] Song H.J., You S., Jia X.H., Yang J. (2015). MoS_2_ nanosheets decorated with magnetic Fe_3_O_4_ nanoparticles and their ultrafast adsorption for wastewater treatment. Ceram. Int..

[187] Li H., Xie F., Li W., Fahlman B.D., Chen M., Li W. (2016). Preparation and adsorption capacity of porous MoS_2_ nanosheets. RSC Adv..

[188] Massey A.T., Gusain R., Kumari S., Khatri O.P. (2016). Hierarchical microspheres of MoS_2_ nanosheets: Efficient and regenerative adsorbent for removal of water-soluble dyes. Ind. Eng. Chem. Res..

[189] Krishna Kumar A.S., Jiang S.J., Warchoł J.K. (2017). Synthesis and characterization of two-dimensional transition metal dichalcogenide magnetic MoS_2_@Fe_3_O_4_ nanoparticles for adsorption of Cr(VI)/Cr(III). ACS Omega.

[190] Wan Z., Li D., Jiao Y., Ouyang X., Chang L., Wang X. (2017). Bifunctional MoS_2_ coated melamine-formaldehyde sponges for efficient oil–water separation and water-soluble dye removal. Appl. Mater. Today.

[191] Li R., Deng H., Zhang X., Wang J.J., Awasthi M.K., Wang Q., Xiao R., Zhou B., Du J., Zhang Z. (2019). High-efficiency removal of Pb(II) and humate by a CeO_2_–MoS_2_ hybrid magnetic biochar. Bioresour. Technol..

[192] Wan Z., Liu Y., Chen S., Song K., Peng Y., Zhao N., Ouyang X., Wang X. (2018). Facile fabrication of a highly durable and flexible MoS_2_@RTV sponge for efficient oil-water separation. Colloids Surfaces A Physicochem. Eng. Asp..

[193] Heiranian M., Farimani A.B., Aluru N.R. (2015). Water desalination with a single-layer MoS_2_ nanopore. Nat. Commun..

[194] Kou J., Yao J., Wu L., Zhou X., Lu H., Wu F., Fan J. (2016). Nanoporous two-dimensional MoS_2_ membranes for fast saline solution purification. Phys. Chem. Chem. Phys..

[195] Kozubek R., Tripathi M., Ghorbani-Asl M., Kretschmer S., Madauß L., Pollmann E., O’Brien M., McEvoy N., Ludacka U., Susi T. (2019). Perforating freestanding molybdenum disulfide monolayers with highly charged ions. J. Phys. Chem. Lett..

[196] Ma J., Tang X., He Y., Fan Y., Chen J., Hao Y. (2020). Robust stable MoS_2_/GO filtration membrane for effective removal of dyes and salts from water with enhanced permeability. Desalination.

[197] Gao J., Zhang M., Wang J., Liu G., Liu H., Jiang Y. (2019). Bioinspired modification of layer-stacked molybdenum disulfide (MoS_2_) membranes for enhanced nanofiltration performance. ACS Omega.

[198] Chu H., Liu X., Liu B., Zhu G., Lei W., Du H., Liu J., Li J., Li C., Sun C. (2016). Hexagonal 2H-MoSe_2_ broad spectrum active photocatalyst for Cr(VI) reduction. Sci. Rep..

[199] Mittal H., Khanuja M. (2019). Nanosheets- and nanourchins-like nanostructures of MoSe_2_ for photocatalytic water purification: Kinetics and reusability study. Environ. Sci. Pollut. Res..

[200] Zhou X., Yao J., Yang M., Ma J., Zhou Q., Ou E., Zhang Z., Sun X. (2018). Synthesis of MoSe_2_/SrTiO_3_ heterostructures with enhanced ultraviolet-light-driven and visible-light-driven photocatalytic properties. Nano.

[201] Kridiotis A.C., Longwell J.P., Sarofim A.F., Bar-ziv E. (1989). Application of a stochastic model of imperfect mixing to the combustion of fuel-lean COH_2_ mixtures in air. Chem. Eng. Sci..

[202] Kim J.H., Ma X., Zhou A., Song C. (2006). Ultra-deep desulfurization and denitrogenation of diesel fuel by selective adsorption over three different adsorbents: A study on adsorptive selectivity and mechanism. Catal. Today.

[203] Kalam M.A., Masjuki H.H. (2004). Emissions and deposit characteristics of a small diesel engine when operated on preheated crude palm oil. Biomass Bioenergy.

[204] Ashraful A.M., Masjuki H.H., Kalam M.A., Rizwanul Fattah I.M., Imtenan S., Shahir S.A., Mobarak H.M. (2014). Production and comparison of fuel properties, engine performance, and emission characteristics of biodiesel from various non-edible vegetable oils: A review. Energy Convers. Manag..

[205] Song C., Ma X. (2003). New design approaches to ultra-clean diesel fuels by deep desulfurization and deep dearomatization. Appl. Catal. B Environ..

[206] Itthibenchapong V., Srifa A., Kaewmeesri R., Kidkhunthod P., Faungnawakij K. (2017). Deoxygenation of palm kernel oil to jet fuel-like hydrocarbons using Ni-MoS_2_/γ-Al_2_O_3_ catalysts. Energy Convers. Manag..

[207] Jiang L., Kronbak J., Christensen L.P. (2014). The costs and benefits of sulphur reduction measures: Sulphur scrubbers versus marine gas oil. Transp. Res. Part D Transp. Environ..

[208] Ushakov S., Valland H., Nielsen J.B., Hennie E. (2013). Effects of high sulphur content in marine fuels on particulate matter emission characteristics. J. Mar. Eng. Technol..

[209] Jones J., Dupont V., Brydson R., Fullerton D., Nasri N., Ross A., Westwood A.V. (2003). Sulphur poisoning and regeneration of precious metal catalysed methane combustion. Catal. Today.

[210] Paul J.F., Payen E. (2003). Vacancy formation on MoS_2_ hydrodesulfurization catalyst: DFT study of the mechanism. J. Phys. Chem. B.

[211] Grimblot J. (1998). Genesis, architecture and nature of sites of Co(Ni)-MoS_2_ supported hydroprocessing catalysts. Catal. Today.

[212] Liaw S.J., Raje A., Chary K.V.R., Davis B.H. (1995). Catalytic hydrotreatment of Illinois No. 6 coal-derived naphtha: The removal of individual nitrogen and sulfur compounds over MoS_2_ and Mo_2_N. Appl. Catal. A Gen..

[213] Mahmoudabadi Z.S., Rashidi A., Tavasoli A. (2020). Synthesis of MoS_2_ quantum dots as a nanocatalyst for hydrodesulfurization of naphtha: Experimental and DFT study. J. Environ. Chem. Eng..

[214] Olivas A., Zepeda T.A., Villalpando I., Fuentes S. (2008). Performance of unsupported Ni(Co,Fe)/MoS_2_ catalysts in hydrotreating reactions. Catal. Commun..

[215] Liu L.H., Liu D., Liu B., Li G.C., Liu Y.Q., Liu C.G. (2011). Relation between the morphology of MoS_2_ in NiMo catalyst and its selectivity for dibenzothiophene hydrodesulfurization. Ranliao Huaxue Xuebao J. Fuel Chem. Technol..

[216] Rangarajan S., Mavrikakis M. (2017). On the preferred active sites of promoted MoS_2_ for hydrodesulfurization with minimal organonitrogen inhibition. ACS Catal..

[217] Tye C.T., Smith K.J. (2006). Hydrodesulfurization of dibenzothiophene over exfoliated MoS_2_ catalyst. Catal. Today.

[218] Abbasi A., Karimi A., Aghabozorg H., Sadighi S., Aval M.A. (2020). Cobalt-promoted MoS_2_ nanosheets: A promising novel diesel hydrodesulfurization catalyst. Int. J. Chem. Kinet..

[219] Moses P.G., Hinnemann B., Topsøe H., Nørskov J.K. (2007). The hydrogenation and direct desulfurization reaction pathway in thiophene hydrodesulfurization over MoS_2_ catalysts at realistic conditions: A density functional study. J. Catal..

[220] Kaluža L., Zdražil M., Žilková N., Čejka J. (2002). High activity of highly loaded MoS_2_ hydrodesulfurization catalysts supported on organised mesoporous alumina. Catal. Commun..

[221] Liu N., Wang X., Xu W., Hu H., Liang J., Qiu J. (2014). Microwave-assisted synthesis of MoS_2_/graphene nanocomposites for efficient hydrodesulfurization. Fuel.

[222] Yang L., Wang X.z., Liu Y., Yu Z.f., Liang J.j., Chen B.b., Shi C., Tian S., Li X., Qiu J.-S. (2017). Monolayer MoS_2_ anchored on reduced graphene oxide nanosheets for efficient hydrodesulfurization. Appl. Catal. B Environ..

[223] Liu G., Robertson A.W., Li M.M.J., Kuo W.C.H., Darby M.T., Muhieddine M.H., Lin Y.C., Suenaga K., Stamatakis M., Warner J.H. (2017). MoS_2_ monolayer catalyst doped with isolated Co atoms for the hydrodeoxygenation reaction. Nat. Chem..

[224] Li Q., Bai X., Ling C., Zhou Q., Yuan S., Chen Q., Wang J. (2019). Forming atom–vacancy interface on the MoS_2_ catalyst for efficient hydrodeoxygenation reactions. Small Methods.

[225] Wu K., Wang W., Guo H., Yang Y., Huang Y., Li W., Li C. (2020). Engineering co nanoparticles supported on defect MoS_2–x_ for mild deoxygenation of lignin-derived phenols to arenes. ACS Energy Lett..

[226] Varakin A.N., Mozhaev A.V., Pimerzin A.A., Nikulshin P.A. (2019). Toward HYD/DEC selectivity control in hydrodeoxygenation over supported and unsupported Co(Ni)-MoS_2_ catalysts. A key to effective dual-bed catalyst reactor for co-hydroprocessing of diesel and vegetable oil. Catal. Today.

[227] Alvarez-Galvan M., Campos-Martin J., Fierro J. (2019). Transition metal phosphides for the catalytic hydrodeoxygenation of waste oils into green diesel. Catalysts.

[228] Li N., Wei L., Bibi R., Chen L., Liu J., Zhang L., Zheng Y., Zhou J. (2016). Catalytic hydrogenation of alkali lignin into bio-oil using flower-like hierarchical MoS_2_-based composite catalysts. Fuel.

[229] Zhou X., Zhou J., Yu Y., Ma J., Sun X., Hu L. (2017). Catalytic hydrogenation of alkali lignin into bio-oil via nano-lamellar MoSe_2_-based composite catalysts. Nano.

[230] Porsin A.A., Vlasova E.N., Bukhtiyarova G.A., Nuzhdin A.L., Bukhtiyarov V.I. (2018). Sulfide catalysts for production of motor fuels from fatty acid triglycerides. Russ. J. Appl. Chem..

[231] Zhang Y., Meng Z., Shi Q., Gao H., Liu Y., Wang Y., Rao D., Deng K., Lu R. (2017). Nanoporous MoS_2_ monolayer as a promising membrane for purifying hydrogen and enriching methane. J. Phys. Condens. Matter.

[232] Yu L., Mishra I.K., Xie Y., Zhou H., Sun J., Zhou J., Ni Y., Luo D., Yu F., Yu Y. (2018). Ternary Ni_2(1−x)_Mo_2x_P nanowire arrays toward efficient and stable hydrogen evolution electrocatalysis under large-current-density. Nano Energy.

[233] Mohamed M., Yusup S., Loy A.C.M. (2019). Effect of empty fruit bunch in calcium oxide for cyclic CO_2_ capture. Chem. Eng. Technol..

[234] Yamamoto A., Shinkai T., Loy A.C.M., Mohamed M., Balean F.H., Yusup S., Quitain A.T., Kida T. (2020). Application of a solid electrolyte CO_2_ sensor to the performance evaluation of CO_2_ capture materials. Sensors Actuators B Chem..

[235] Holloway S. (2005). Underground sequestration of carbon dioxide—A viable greenhouse gas mitigation option. Energy.

[236] Song Y., Peng R., Hensley D.K., Bonnesen P.V., Liang L., Wu Z., Meyer H.M., Chi M., Ma C., Sumpter B.G. (2016). High-selectivity electrochemical conversion of CO_2_ to ethanol using a copper nanoparticle/N-doped graphene electrode. ChemistrySelect.

[237] Yang X., Zhang R., Fu J., Geng S., Cheng J.J., Sun Y. (2014). Pyrolysis kinetic and product analysis of different microalgal biomass by distributed activation energy model and pyrolysis-gas chromatography-mass spectrometry. Bioresour. Technol..

[238] Manenti F. (2015). syngas. Computer Aided Chemical Engineering.

[239] Marlin D.S., Sarron E., Sigurbjörnsson Ó. (2018). Process advantages of direct CO_2_ to methanol synthesis. Front. Chem..

[240] Melo Bravo P., Debecker D.P. (2019). Combining CO_2_ capture and catalytic conversion to methane. Waste Dispos. Sustain. Energy.

[241] Nguyen D.L.T., Kim Y., Hwang Y.J., Won D.H. (2020). Progress in development of electrocatalyst for CO_2_ conversion to selective CO production. Carbon Energy.

[242] Ali N., Bilal M., Nazir M.S., Khan A., Ali F., Iqbal H.M.N. (2020). Thermochemical and electrochemical aspects of carbon dioxide methanation: A sustainable approach to generate fuel via waste to energy theme. Sci. Total Environ..

[243] Wang Z., Roberts R.R., Naterer G.F., Gabriel K.S. (2012). Comparison of thermochemical, electrolytic, photoelectrolytic and photochemical solar-to-hydrogen production technologies. Int. J. Hydrogen Energy.

[244] Zhang J.-H., Zhou Y.-G. (2020). Nano-impact electrochemistry: Analysis of single bioentities. TrAC Trends Anal. Chem..

[245] Abdullah H., Khan M.M.R., Ong H.R., Yaakob Z. (2017). Modified TiO_2_ photocatalyst for CO_2_ photocatalytic reduction: An overview. J. CO2 Util..

[246] Xie S., Zhang Q., Liu G., Wang Y. (2016). Photocatalytic and photoelectrocatalytic reduction of CO_2_ using heterogeneous catalysts with controlled nanostructures. Chem. Commun..

[247] Kisch H. (2013). Semiconductor photocatalysis-mechanistic and synthetic aspects. Angew. Chemie Int. Ed..

[248] Xu Q., Zhang L., Yu J., Wageh S., Al-Ghamdi A.A., Jaroniec M. (2018). Direct Z-scheme photocatalysts: Principles, synthesis, and applications. Mater. Today.

[249] Geioushy R.A., El-Sheikh S.M., Hegazy I.M., Shawky A., El-Sherbiny S., Kandil A.H.T. (2019). Insights into two-dimensional MoS_2_ sheets for enhanced CO_2_ photoreduction to C1 and C2 hydrocarbon products. Mater. Res. Bull..

[250] Beheshti M., Kakooei S., Ismail M.C., Shahrestani S. (2020). Investigation of CO_2_ electrochemical reduction to syngas on Zn/Ni-based electrocatalysts using the cyclic voltammetry method. Electrochim. Acta.

[251] Debe M.K. (2012). Electrocatalyst approaches and challenges for automotive fuel cells. Nature.

[252] Zinola C.F., Martins M.E., Tejera E.P., Neves N.P. (2012). Electrocatalysis: Fundamentals and applications. Int. J. Electrochem..

[253] Asadi M., Kumar B., Behranginia A., Rosen B.A., Baskin A., Repnin N., Pisasale D., Phillips P., Zhu W., Haasch R. (2014). Robust carbon dioxide reduction on molybdenum disulphide edges. Nat. Commun..

[254] Wang Y., Zhang Z., Zhang L., Luo Z., Shen J., Lin H., Long J., Wu J.C.S., Fu X., Wang X. (2018). Visible-light driven overall conversion of CO_2_ and H_2_O to CH_4_ and O_2_ on 3D-SiC@2D-MoS_2_ Heterostructure. J. Am. Chem. Soc..

[255] Asadi M., Kim K., Liu C., Addepalli A.V., Abbasi P., Yasaei P., Phillips P., Behranginia A., Cerrato J.M., Haasch R. (2016). Nanostructured transition metal dichalcogenide electrocatalysts for CO_2_ reduction in ionic liquid. Science.

[256] Lee H.I., Yu H., Rhee C.K., Sohn Y. (2019). Electrochemical hydrogen evolution and CO_2_ reduction over hierarchical MoS_x_Se_2−x_ hybrid nanostructures. Appl. Surf. Sci..

[257] Xie Y., Li X., Wang Y., Li B., Yang L., Zhao N., Liu M., Wang X., Yu Y., Liu J.M. (2020). Reaction mechanisms for reduction of CO_2_ to CO on monolayer MoS_2_. Appl. Surf. Sci..

[258] Abbasi P., Asadi M., Liu C., Sharifi-Asl S., Sayahpour B., Behranginia A., Zapol P., Shahbazian-Yassar R., Curtiss L.A., Salehi-Khojin A. (2017). Tailoring the edge structure of molybdenum disulfide toward electrocatalytic reduction of carbon dioxide. ACS Nano.

[259] Asadi M., Motevaselian M.H., Moradzadeh A., Majidi L., Esmaeilirad M., Sun T.V., Liu C., Bose R., Abbasi P., Zapol P. (2019). Highly efficient solar-driven carbon dioxide reduction on molybdenum disulfide catalyst using choline chloride-based electrolyte. Adv. Energy Mater..

[260] Liu X., Yang H., He J., Liu H., Song L., Li L., Luo J. (2018). Highly active, durable ultrathin MoTe_2_ layers for the electroreduction of CO_2_ to CH_4_. Small.

[261] Primo A., He J., Jurca B., Cojocaru B., Bucur C., Parvulescu V.I., Garcia H. (2019). CO_2_ methanation catalyzed by oriented MoS_2_ nanoplatelets supported on few layers graphene. Appl. Catal. B Environ..

[262] Jung H., Cho K.M., Kim K.H., Yoo H.W., Al-Saggaf A., Gereige I., Jung H.T. (2018). Highly efficient and stable CO_2_ reduction photocatalyst with a hierarchical structure of mesoporous TiO_2_ on 3D graphene with few-layered MoS_2_. ACS Sustain. Chem. Eng..

[263] Qin H., Guo R.T., Liu X.Y., Pan W.G., Wang Z.Y., Shi X., Tang J.Y., Huang C.Y. (2018). Z-scheme MoS_2_/g-C_3_N_4_ heterojunction for efficient visible light photocatalytic CO_2_ reduction. Dalt. Trans..

[264] Jia P.y., Guo R.t., Pan W.g., Huang C.y., Tang J.y., Liu X.y., Qin H., Xu Q.Y. (2019). The MoS_2_/TiO_2_ heterojunction composites with enhanced activity for CO_2_ photocatalytic reduction under visible light irradiation. Colloids Surfaces A Physicochem. Eng. Asp..

[265] Kanan M.W., Nocera D.G. (2008). In situ formation of an oxygen-evolving catalyst in neutral water containing phosphate and Co^2+^. Science.

[266] Reece S.Y., Hamel J.A., Sung K., Jarvi T.D., Esswein A.J., Pijpers J.J.H., Nocera D.G. (2011). Wireless solar water splitting using silicon-based semiconductors and earth-abundant catalysts. Science.

[267] Wang Q., Lei Y., Wang D., Li Y. (2019). Defect engineering in earth-abundant electrocatalysts for CO_2_ and N_2_ reduction. Energy Environ. Sci..

[268] Ji Y., Nørskov J.K., Chan K. (2019). Scaling relations on basal plane vacancies of transition metal dichalcogenides for CO_2_ reduction. J. Phys. Chem. C.

[269] Pérez-Fortes M., Schöneberger J.C., Boulamanti A., Tzimas E. (2016). Methanol synthesis using captured CO_2_ as raw material: Techno-economic and environmental assessment. Appl. Energy.

[270] Pérez-Fortes M., Bocin-Dumitriu A., Tzimas E. (2014). CO_2_ utilization pathways: Techno-economic assessment and market opportunities. Energy Procedia.

[271] Zheng Y., Yin X., Jiang Y., Bai J., Tang Y., Shen Y., Zhang M. (2019). Nano Ag-decorated MoS_2_ nanosheets from 1T to 2H phase conversion for photocatalytically reducing CO_2_ to methanol. Energy Technol..

[272] Bolivar H., Izquierdo S., Tremont R., Cabrera C.R. (2003). Methanol oxidation at Pt/MoO_x_/MoSe_2_ thin film electrodes prepared with exfoliated MoSe_2_. J. Appl. Electrochem..

[273] Tu W., Li Y., Kuai L., Zhou Y., Xu Q., Li H., Wang X., Xiao M., Zou Z. (2017). Construction of unique two-dimensional MoS_2_-TiO_2_ hybrid nanojunctions: MoS_2_ as a promising cost-effective cocatalyst toward improved photocatalytic reduction of CO_2_ to methanol. Nanoscale.

[274] Xu F., Zhu B., Cheng B., Yu J., Xu J. (2018). 1D/2D TiO_2_/MoS_2_ hybrid nanostructures for enhanced photocatalytic CO_2_ reduction. Adv. Opt. Mater..

[275] Francis S.A., Velazquez J.M., Ferrer I.M., Torelli D.A., Guevarra D., McDowell M.T., Sun K., Zhou X., Saadi F.H., John J. (2018). Reduction of aqueous CO_2_ to 1-propanol at MoS_2_ electrodes. Chem. Mater..

[276] Dai W., Yu J., Luo S., Hu X., Yang L., Zhang S., Li B., Luo X., Zou J. (2020). WS_2_ quantum dots seeding in Bi_2_S_3_ nanotubes: A novel Vis-NIR light sensitive photocatalyst with low-resistance junction interface for CO_2_ reduction. Chem. Eng. J..

[277] Dai W., Yu J., Deng Y., Hu X., Wang T., Luo X. (2017). Facile synthesis of MoS_2_/Bi_2_ WO_6_ nanocomposites for enhanced CO_2_ photoreduction activity under visible light irradiation. Appl. Surf. Sci..

[278] Peng H., Lu J., Wu C., Yang Z., Chen H., Song W., Li P., Yin H. (2015). Co-doped MoS_2_ NPs with matched energy band and low overpotential high efficiently convert CO_2_ to methanol. Appl. Surf. Sci..

[279] Biswas M.R.U.D., Ali A., Cho K.Y., Oh W.C. (2018). Novel synthesis of WSe_2_-Graphene-TiO_2_ ternary nanocomposite via ultrasonic technics for high photocatalytic reduction of CO_2_ into CH_3_OH. Ultrason. Sonochem..

[280] Reyes Valle C., Villanueva Perales A.L., Vidal-Barrero F., Gómez-Barea A. (2013). Techno-economic assessment of biomass-to-ethanol by indirect fluidized bed gasification: Impact of reforming technologies and comparison with entrained flow gasification. Appl. Energy.

[281] Villanueva Perales A.L., Reyes Valle C., Ollero P., Gómez-Barea A. (2011). Technoeconomic assessment of ethanol production via thermochemical conversion of biomass by entrained flow gasification. Energy.

[282] Phillips S.D. (2007). Technoeconomic analysis of a lignocellulosic biomass indirect gasification process to make ethanol via mixed alcohols synthesis. Ind. Eng. Chem. Res..

[283] Kwok K.M., Ong S.W.D., Chen L., Zeng H.C. (2018). Constrained growth of MoS_2_ nanosheets within a mesoporous silica shell and its effects on defect sites and catalyst stability for H_2_S decomposition. ACS Catal..

[284] Xi J., Zhao T., Wang D., Shuai Z. (2014). Tunable electronic properties of two-dimensional transition metal dichalcogenide alloys: A first-principles prediction. J. Phys. Chem. Lett..

[285] Ahmed S., Yi J. (2017). Two-dimensional transition metal dichalcogenides and their charge carrier mobilities in field-effect transistors. Nano Micro Lett..

[286] Cates E.L. (2017). Photocatalytic water treatment: So where are we going with this?. Environ. Sci. Technol..

[287] Hodges B.C., Cates E.L., Kim J.H. (2018). Challenges and prospects of advanced oxidation water treatment processes using catalytic nanomaterials. Nat. Nanotechnol..

[288] Chen X., Liu C., Mao S. (2020). Environmental analysis with 2D transition-metal dichalcogenide-based field-effect transistors. Nano Micro Lett..

[289] Ngan S.L., How B.S., Teng S.Y., Leong W.D., Loy A.C.M., Yatim P., Promentilla M.A.B., Lam H.L. (2020). A hybrid approach to prioritize risk mitigation strategies for biomass polygeneration systems. Renew. Sustain. Energy Rev..

[290] Vondra M., Touš M., Teng S.Y. (2019). Digestate evaporation treatment in biogas plants: A Techno-economic assessment by Monte Carlo, neural networks and decision trees. J. Clean. Prod..

[291] Teng S.Y., How B.S., Leong W.D., Teoh J.H., Siang Cheah A.C., Motavasel Z., Lam H.L. (2019). Principal component analysis-aided statistical process optimisation (PASPO) for process improvement in industrial refineries. J. Clean. Prod..

[292] Xu C., Gao S., Li M. (2017). A novel PCA-based microstructure descriptor for heterogeneous material design. Comput. Mater. Sci..

[293] Teng S.Y., Loy A.C.M., Leong W.D., How B.S., Chin B.L.F., Máša V. (2019). Catalytic thermal degradation of *Chlorella vulgaris*: Evolving deep neural networks for optimization. Bioresour. Technol..

[294] Ge L., Yuan H., Min Y., Li L., Chen S., Xu L., Goddard W.A. (2020). Predicted optimal bifunctional electrocatalysts for the hydrogen evolution reaction and the oxygen evolution reaction using chalcogenide heterostructures based on machine learning analysis of in silico quantum mechanics based high throughput screening. J. Phys. Chem. Lett..

[295] Zhao H., Hu R., Xie X., Wang Z., Jiang P., Zheng C., Gao X., Wu T. (2018). Adopting big data to accelerate discovery of 2D TMDCs materials via CVR method for the potential application in urban airborne Hg^0^ sensor. Energy Procedia.

[296] Bassman L., Rajak P., Kalia R.K., Nakano A., Sha F., Aykol M., Huck P., Persson K., Sun J., Singh D.J. (2018). Efficient discovery of optimal *N*-layered TMDC hetero-structures. MRS Adv..

